# Structural insights into endogenous agonist selectivity of aminergic receptors from the octopamine β_2_ receptor

**DOI:** 10.1093/pnasnexus/pgaf376

**Published:** 2025-11-28

**Authors:** Tetsuya Hori, Kazushige Katsura, Sayako Miyamoto-Kohno, Tomomi Uchikubo-Kamo, Mayumi Yonemochi, Mikako Shirouzu

**Affiliations:** Laboratory for Protein Functional and Structural Biology, RIKEN Center for Integrative Medical Sciences, 1-7-22 Suehiro-cho, Tsurumi-ku, Yokohama, Kanagawa 230-0045, Japan; Laboratory for Protein Functional and Structural Biology, RIKEN Center for Integrative Medical Sciences, 1-7-22 Suehiro-cho, Tsurumi-ku, Yokohama, Kanagawa 230-0045, Japan; Drug Discovery Structural Biology Platform Unit, RIKEN Center for Integrative Medical Sciences, 1-7-22 Suehiro-cho, Tsurumi-ku, Yokohama, Kanagawa 230-0045, Japan; Drug Discovery Structural Biology Platform Unit, RIKEN Center for Integrative Medical Sciences, 1-7-22 Suehiro-cho, Tsurumi-ku, Yokohama, Kanagawa 230-0045, Japan; Laboratory for Protein Functional and Structural Biology, RIKEN Center for Integrative Medical Sciences, 1-7-22 Suehiro-cho, Tsurumi-ku, Yokohama, Kanagawa 230-0045, Japan; Drug Discovery Structural Biology Platform Unit, RIKEN Center for Integrative Medical Sciences, 1-7-22 Suehiro-cho, Tsurumi-ku, Yokohama, Kanagawa 230-0045, Japan; Drug Discovery Structural Biology Platform Unit, RIKEN Center for Integrative Medical Sciences, 1-7-22 Suehiro-cho, Tsurumi-ku, Yokohama, Kanagawa 230-0045, Japan; Laboratory for Protein Functional and Structural Biology, RIKEN Center for Integrative Medical Sciences, 1-7-22 Suehiro-cho, Tsurumi-ku, Yokohama, Kanagawa 230-0045, Japan; Drug Discovery Structural Biology Platform Unit, RIKEN Center for Integrative Medical Sciences, 1-7-22 Suehiro-cho, Tsurumi-ku, Yokohama, Kanagawa 230-0045, Japan; Structural Life Science and Cell Biology Collaboration Team, RIKEN Center for Biosystems Dynamics Research, 1-7-22 Suehiro-cho, Tsurumi-ku, Yokohama, Kanagawa 230-0045, Japan

**Keywords:** GRCR, cryo-EM, amine ligand selectivity, acaricide

## Abstract

Tyrosine-derived amines (TDAs), such as octopamine, noradrenaline, dopamine, and tyramine, are essential neurotransmitters that play diverse roles in various physiological processes. The distinct receptor selectivity of these structurally similar molecules is vital for their specific functions. However, all these receptors belong to the same subfamily of G-protein-coupled receptors and share high sequence homology within their orthosteric binding sites. The molecular basis of this selectivity remains unclear because of the absence of structural data on octopamine and tyramine receptors. In this study, we present cryo-electron microscopy structures of the deer tick octopamine β_2_ receptor (octβ_2_R) bound to octopamine or *N*-2,4-dimethylphenyl-*N*′-methylformamidine (DPMF), an acaricidal amitraz metabolite. Octopamine and DPMF formed aromatic interactions with Y307^6.55^ and F328^7.39^, residues crucial for octβ_2_R activation. The lower potency of other TDAs for octβ_2_R stems from the subtle effect of functional groups on both interactions, i.e. the meta-hydroxyl group of noradrenaline and dopamine hinders edge-to-edge interaction with Y307^6.55^, and the absence of a 1-hydroxyl group in dopamine and tyramine prevents π-hydrogen bonding with F328^7.39^. These structural insights into octβ_2_R selectivity are likely applicable across other TDA receptors, highlighting the pivotal role of residues 6.55 and 7.39. Consequently, the elucidated selection mechanism provides fundamental knowledge of aminergic ligand recognition, a process crucial for neurotransmission and overall organismal function.

Significance Statement Tyrosine-derived hormones, including noradrenaline, dopamine, octopamine, and tyramine, exhibit distinct receptor selectivity despite their structural similarities; the molecular mechanisms of this selectivity remain unclear. The structure of the octopamine β_2_ receptor and octopamine complex indicates that subtle variations in aromatic and/or van der Waals interactions with unique residues at positions 6.55 and 7.39 play a key role in this selectivity. This structural insight provides a foundation for understanding ligand selectivity across these receptors, facilitating the development of selective agonists with minimized side effects for adrenaline and dopamine receptors and an effective acaricide for the octopamine receptor.

## Introduction

Octopamine—a tyrosine-derived amine (TDA)—is structurally similar to tyramine, noradrenaline, and dopamine. It functions as an invertebrate hormone and neurotransmitter and regulates various physiological effects (Fig. S1), including the modulation of feeding rate, satiety, food selection, appetite, locomotion, physical activity, obesity, metabolic rate, and sleep. Its effects closely resemble those of noradrenaline and adrenaline in vertebrates (1, 2).

Octopaminergic neurons regulate their effects by activating octopamine receptors. Octβ_2_R, an aminergic G-protein-coupled receptor (GPCR), is one of the major octopamine receptor subtypes. Although three octβRs (octβ_1_R, octβ_2_R, and octβ_3_R) have been identified in certain invertebrates, such as *Drosophila*, only octβ_2_R has been characterized in ticks and mites. The octopamine-octβ_2_R signaling inhibits oviduct contractions, thereby facilitating egg release (3, 4). Additionally, octβ2R has been implicated in locomotion activity (5) and feeding behavior (6). High octβ2R expression in male antennae and beaks indicates its potential role in sensory perception, including olfaction, gustation, and possibly hearing (7). Moreover, octβ2R is highly expressed in the fat body, a significant metabolic organ (8, 9).

All TDA receptors belong to class-A GPCRs. Adrenergic receptors (ARs) and dopamine receptors (DRs), in complex with their endogenous agonists, exhibit similar 3D structures (10–16). The residues forming their orthosteric binding sites, including those for octopamine and tyramine receptors (TARs), are highly conserved, except for three residues—45.52 (at extracellular loop [ECL] 2), 6.55, and 7.39 (Ballesteros–Weinstein numbering for GPCR (17)) (Table S1). Although the binding modes of each endogenous agonist at ARs and DRs have been elucidated, the molecular mechanism explaining why TDAs exhibit lower potency for a given receptor than their endogenous agonists remains unclear.

Amitraz, an acaricide first synthesized in 1969 (18), is widely used to control ticks and mites infesting various hosts, including dogs, bees, and plants (19, 20). It exhibits low toxicity to host organisms, likely because of its efficient detoxification mechanisms or controlled drug delivery systems (21). Following amitraz treatment, ticks and mites rapidly detach from their hosts owing to excessive stimulation (22, 23), often resulting in feeding and mating abnormalities and eventual death (23). Amitraz is a prodrug that undergoes rapid metabolic conversion to *N*2-(2,4-dimethylphenyl)-*N*1-methyformamidine (DPMF) and 2′,4′-formxylidine (*N*-(2,4-dimethylphenyl)formamide) (24) (Fig. S2a). Although both amitraz and DPMF act as agonists at various octopamine-related receptors in vitro—including octαR (homologous to the octopamine receptor from mushroom body [OAMB], α_1_AR, and α_2_AR), octβ_2_R, and TAR across different species—the acaricidal effect of amitraz is primarily attributed to the agonistic action of DPMF at octβ_2_R in ticks and mites (25). Resistance to amitraz associated with mutations in octβ_2_R has been reported in certain acarids, including the cattle tick (*Rhipicephalus microplus*) and varroa mite (*Varroa destructor*) (20, 22, 26, 27). The molecular mechanism of DPMF binding and amitraz resistance in octβ_2_R remains unclear.

To address these gaps, we present cryo-electron microscopy (cryo-EM) structures of the deer tick *Ixodes scapularis* octβ_2_R (is-octβ_2_R) and Gs-protein complex, with bound DPMF or octopamine. These structures elucidate the molecular determinants of agonist binding, providing insights into the mechanisms of agonist action and the molecular basis of DPMF resistance observed in the mutant receptor. Additionally, our findings explain why TDAs that are not endogenous agonists for each aminergic GPCR exhibit lower binding affinities for their receptors.

## Results

### Structure of octβ_2_R and G-protein complex

**Fig. 1.**
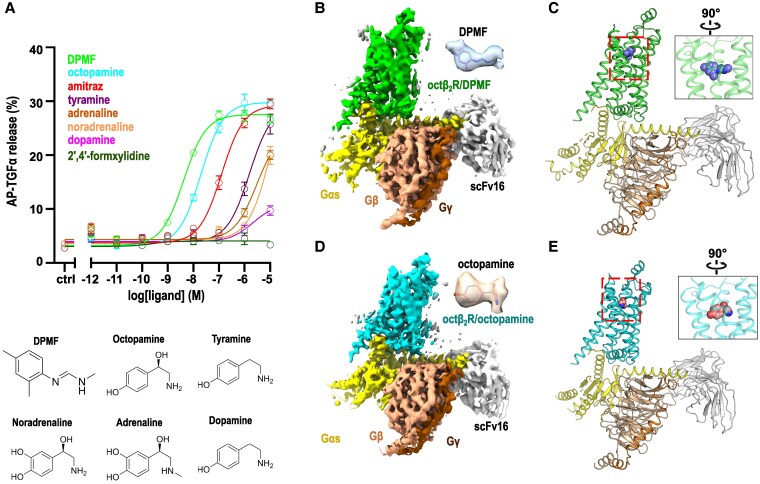
Functional assay of *I. scapularis* octβ_2_R (is-octβ_2_R) and agonist complexes. A) Concentration-response curves of the endogenous TDAs and amitraz-related compounds to is-octβ_2_R by a TGFα shedding assay. Data are presented as the mean ± standard error of the mean from three independent experiments (*n* = 3). A summary of EC_50_ and *E*_max_ values is presented in Table S2. B–E) Cryo-EM maps and structures of is-octβ_2_R/DPMF (B and C) and is-octβ_2_R/oct (d and e). The orthosteric binding site is denoted by a rectangle. An enlarged orthosteric binding site viewed from the left side of is-octβ_2_R is demonstrated.

Before structural analysis, we characterized the pharmacological properties of *I. scapularis* octβ_2_R (is-octβ_2_R) using a tumor growth factor-alpha (TGFα) shedding assay (28) (Fig. 1A and Table S2). This assay assessed the potencies of various ligands, including octopamine, amitraz, DPMF, and other TDAs. Octopamine, amitraz, and DPMF exhibited high potency at is-octβ_2_R, with half-maximal effective concentration (EC_50_) values in the 10-nM range. Notably, no agonistic activity was observed for 2′,4′-formxylidine, another amitraz metabolite (Fig. S2a). Although TDAs other than octopamine displayed agonistic activity, their affinities were significantly lower. These affinities align with those reported for octβ_2_R in other species (25, 29, 30).

In this study, we determined two cryo-EM structures of is-octβ_2_R—one bound to DPMF (is-octβ_2_R/DPMF) and the other bound to octopamine (is-octβ_2_R/oct)—with global resolutions of 3.4 and 3.65 Å, respectively (Fig. 1B–E). Both structures were obtained in a high-affinity complex with a nucleotide-free state of is-Gs (*I. scapularis* G-protein α subunit [is-Gαs] and human Gβγ) with apyrase treatment, an enzyme that hydrolyzes guanosine diphosphate (GDP) and guanosine triphosphate (GTP) to guanosine monophosphate (31). The helical domain of the is-Gαs was poorly resolved in either complex, as commonly observed in the nucleotide-free G-protein structures owing to the inherent flexibility of this region.

Structural comparison revealed a high similarity between the is-octβ_2_R/DPMF and is-octβ_2_R/oct complexes. The overall root-mean-square deviation (r.m.s.d.) for all atoms (is-octβ_2_R and G-protein) was 0.71 Å (748 atoms), and the r.m.s.d. for is-octβ_2_R alone was 0.60 Å (249 atoms) (Fig. S3). Because of this significant structural similarity, we primarily used the is-octβ_2_R/DPMF structure for subsequent analyses unless stated otherwise.

is-octβ_2_R is a class-A GPCR that adopts a typical seven-transmembrane (TM7) structure with an additional helix 8 following TM7. The bound DPMF and octopamine occupy nearly identical positions within the central transmembrane (TM) bundle, enclosed by TMs 3, 5, 6, and 7, aligning with the orthosteric binding site commonly observed in class-A GPCRs (32, 33).

The is-octβ_2_R structure closely resembles mammalian aminergic GPCRs, specifically DR1. A distance-matrix alignment (DALI) search identified DR1/SKF83959 (DR1/SKF83959 and Gs complex, PDB ID: 7JVP) as the top hit for both is-octβ_2_R/DPMF and is-octβ2R/oct (34). The r.m.s.d. between DR1/SKF83959 and is-octβ_2_R/DPMF is 0.71 Å for 208 Cα atoms. Notably, all major structural elements, including TM helices and extracellular and intracellular loops (ECLs and ICLs), were closely aligned (Fig. S4b). Phylogenetic analysis indicated that human trace amine-associated receptor 1 (TAAR1) is more closely related to is-octβ_2_R than to DR1 (Fig. S4a). Structurally, is-octβ_2_R/DPMF exhibits a relatively lower similarity to TAAR1 (r.m.s.d. = 0.86 Å for 208 Cα atoms, PDB ID: 8WC8) (Fig. S4c). Although octopamine and noradrenaline share structural similarity, the similarity of is-octβ_2_R to β_1_AR is relatively lower (r.m.s.d. = 0.83 Å for 204 Cα atoms, PDB ID: 7JJO) (Fig. S4d).

### GPCR–G interaction

For structural studies, we used tick is-Gαs. The residues in tick is-Gαs that interact with Gβγ, GDP/GTP, or is-octβ_2_R are the same amino acids as those in human Gαs (Fig. S5). The Ras domain of is-Gαs closely resembles that of human Gαs in DR1/SKF83959 (r.m.s.d. = 1.4 Å, 178 atoms, PDB ID 7JVP). Additionally, the overall structure, including is-octβ_2_R and is-Gs, was similar to that of the DR1 and Gs complexes (r.m.s.d. = 1.3 Å for 704 Cα atoms, PDB ID: 7JVP).

**Fig. 2.**
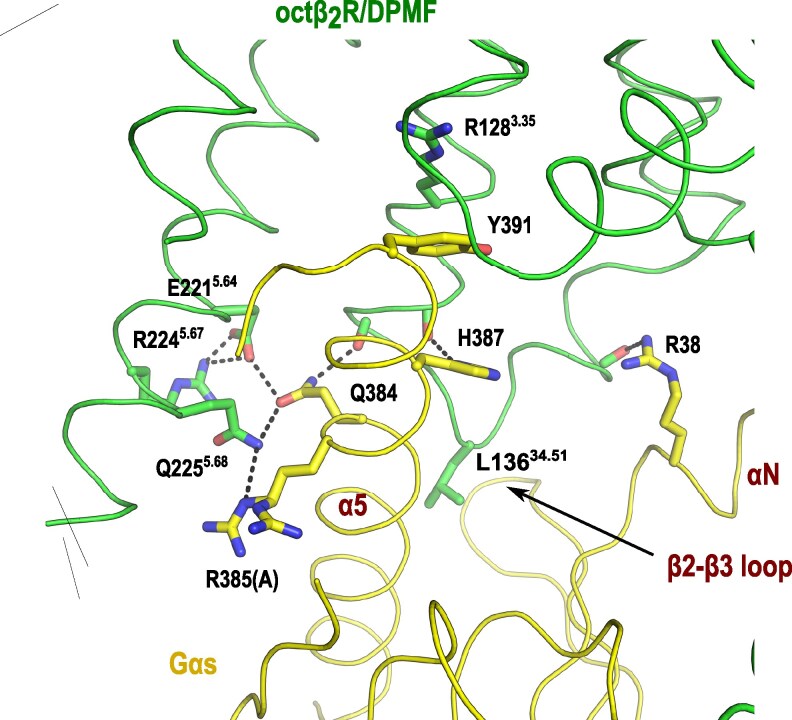
Structure of *I. scapularis* octβ_2_R (is-octβ_2_R)/DPMF and *I. scapularis* G-protein α subunit (is-Gαs) interface. Residues involved in is-octβ_2_R and is-Gαs binding are demonstrated as a stick model. The residue number of is-Gαs is identical to that of human Gαs. The R385 side chain has multiple conformations, and conformation A has an interaction with Q225^5.68^.

The is-Gαs regions interacting with is-octβ_2_R are located in the N- and C-terminal helices (αN and α5) and β2-β3 loop (Figs. 1C, E and 2). The helix α5 of is-Gαs inserts into the is-octβ_2_R helix bundle comprising TMs 2–7 and H8 (Fig. 2). This interface is stabilized by a polar interaction network between TM5 of is-octβ_2_R and the helix α5 of is-Gαs. This involves Q384 and R385 of is-Gαs and E221^5.64^, R224^5.67^, and Q225^5.68^ of is-octβ_2_R. Additionally, hydrogen bonds between the helix α5 of is-Gαs and C-terminus of TM3, specifically between the side chains of Q384 and H387 and the backbone carbonyl oxygens of A131^3.53^ and I132^3.54^, respectively, contribute to complex stability. A characteristic cation–π interaction occurs between Y391 of is-Gαs and R128^3.50^ of is-octβ_2_R, a conserved DRY motif among the class-A GPCRs (31, 35–37) (Fig. 2). Moreover, the helix αN of is-Gαs interacts with the N-terminus of the short α-helix of ICL2 of is-octβ_2_R, specifically between R38 at αN and the backbone carbonyl oxygens of P139^34.54^ at ICL2 (Fig. 2). Furthermore, L136^34.51^ of ICL2 of is-octβ_2_R inserts into a hydrophobic pocket on is-Gαs formed by the αN and α5 helices and the β2-β3 loop (Fig. 2). These interactions closely resemble those observed in other GPCR-Gαs complexes (31, 35).

### Assay and structural consideration of amitraz-resistant mutant

Numerous studies have indicated the global emergence of amitraz-resistant ticks and mites (20). Resistance has been associated with point mutations within the octβ_2_R gene, including T44^1.41^P, I45^1.42^F, I46^1.43^T, or Y72^2.41^S in the cattle tick *R. microplus* from Australia, and N92^2.61^S or Y218^5.58^H in the varroa mite *V. destructor* from France and the United States (20, 22, 26, 27). Notably, all corresponding residues are conserved in is-octβ_2_R and octβ_2_Rs of other species, except for T^1.41^ (Table S3). This study assessed the functional consequences of mutations at these positions in is-octβ_2_R.

**Fig. 3.**
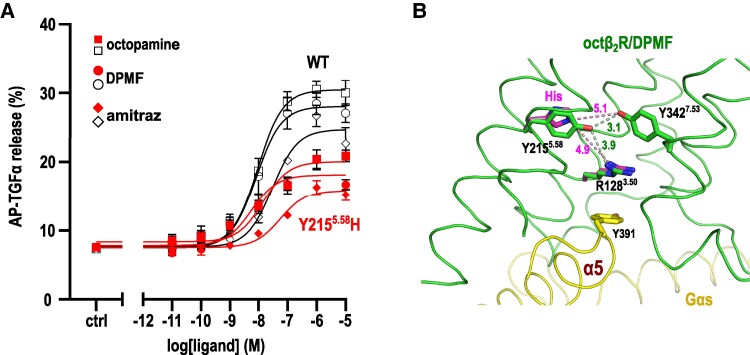
Assay and modeled structure of the amitraz-resistant mutation. A) Results of the TGFα shedding assay for the Y215^5.58^H mutant of *I. scapularis* octβ_2_R (is-octβ_2_R). Data are presented as the mean ± standard error of the mean from three independent experiments (*n* = 3). Results for other mutants and a summary of EC_50_ and *E*_max_ values for all are presented in Fig. S7 and Table S4. B) Structure of is-octβ_2_R/DPMF around Y215^5.58^. Residues involved in the interaction of Y215^5.58^ and Y391 of *I. scapularis* G-protein α subunit are demonstrated as stick models. The side chains of R128^3.50^ and H215^5.58^ of the Y215^5.58^H model are energy-minimized using Phenix and are demonstrated with magenta carbon.

The Y215^5.58^H mutation significantly reduced the maximal response (*E*_max_) to amitraz, DPMF, and octopamine by ∼50% in a cell-based assay (Fig. 3A and Table S4). The EC_50_ for DPMF remained unaffected, whereas those for octopamine and amitraz were reduced by 4- and 6.7-fold, respectively. This reduction in efficacy aligns with previous studies on other class-A GPCRs of the analogous Y^5.58^H mutation (38–41). However, the cell surface expression level of the Y215^5.58^H mutant is slightly decreased compared with the wild type, as observed through immunofluorescence (Fig S6). These findings suggest that the Gi signaling of octopaminergic neurons by the Y^5.58^H mutant in *V. destructor* will be attenuated due to either the functional degradation of the receptor or a reduced expression level of the receptor. In either case, the reduced efficacy of the Y215^5.58^H mutant with DPMF is associated with amitraz resistance in *V. destructor*. In contrast, no significant differences in EC_50_ or *E*_max_ values were observed for the other five mutants (Fig. S7 and Table S4). The I61^1.42^F mutation elicited a result similar to that observed in the octβ_2_R of *R. microplus* using a calcium mobilization assay (25 ). However, the corresponding mutation resulted in a reduced EC_50_ value in the octβ_2_R of the silkworm (*Bombyx mori*) in a cyclic adenosine monophosphate (cAMP) production assay (42). This discrepancy may be attributed to differences in the origin of octβ_2_R or the assay system used.

Residue 5.58 is a highly conserved hydrophilic residue among class-A GPCRs (Table S5). In the is-octβ_2_R structure, the hydroxyl group of Y215^5.58^ formed hydrogen bonds with the side chains of R128^3.50^ and Y342^7.53^, both of which are highly conserved residues within the DRY and NPXXY motifs, respectively (Fig. 3B). The hydrogen-bond network involving Y215^5.58^, R128^3.50^, and Y342^7.53^ likely stabilized the R128^3.50^ position. Additionally, the R128^3.50^ side chain forms a cation–π interaction with Y391 of the Gαs subunit, a characteristic interaction that stabilizes the GPCR and nucleotide-free Gs complex (Fig. 2) (31, 35–37).

The Y215^5.58^H mutation is predicted to disrupt the hydrogen bond network (Fig. 3B), potentially causing the delocalization of the R128^3.50^ side chain without interacting with H215^5.58^ or a rotamer conformation alteration in R128^3.50^ to form a novel hydrogen bond with the shorter side chain of H215^5.58^. Both scenarios may disrupt the crucial cation–π interaction between R128^3.50^ and Y391 of Gαs. However, drawing definitive conclusions based solely on the nucleotide-free Gs-bound complex structure is challenging. First, GPCR-Gs complexes with native N^5.58^ or Q^5.58^ exhibit diverse coupling modes, featuring distinct R128^3.50^ rotamer conformations, loss of the R128^3.50^-Y391 cation–π interaction, and an E^3.49^-Y391 interaction in V2R (PDB ID: 7KH0), EP2 (PDB ID: 7CX2), and EP4 (PDB ID: 7D7 M). Notably, these three GPCRs demonstrate relatively high efficacies compared with others in systematic cell assays (28), indicating that the putative absence of the R^3.50^-Y391 interaction in the nucleotide-free Gs complex may not directly reduce the efficacy of the Y215^5.58^H mutant. Second, the interaction network involving Y^5.58^, R^3.50^, and Y^7.53^ is believed to contribute to the greater structural alterations on the cytoplasmic side of TM6 in Gs-coupled GPCRs compared with those in Gi-coupled GPCRs (43). This indicates that interactions involving R^3.50^, Y^5.58^, and Y^7.53^ may also play a role in earlier stages of GPCR activity before forming the nucleotide-free Gs protein, such as Gs recognition and GDP release. In summary, the reduced efficacy of the Y215^5.58^H mutant likely results from the destabilization of the GPCR-Gs complex in one or more functional states, including before nucleotide-free Gs formation or before Gs coupling (inactive state). Furthermore, the destabilization of octβ_2_R will lead to functional degradation and/or a decrease in the expression level of octβ_2_R.

### DPMF binding mode to is-octβ_2_R

DPMF comprises two moieties: methylformamidine and 2,4-dimethylphenyl (Fig. S2b). The methylformamidine moiety exhibits planarity, and its rotational freedom relative to that of the dimethylphenyl moiety is restricted. This restriction arises from the resonance interactions between the π-electrons of the dimethylphenyl group and lone pair of electrons on the adjacent proximal nitrogen atom. Under physiological conditions, the lone pair of electrons on the proximal nitrogen atom remains unprotonated. The distal nitrogen atom bears a hydrogen and methyl group (Fig. S2).

**Fig. 4.**
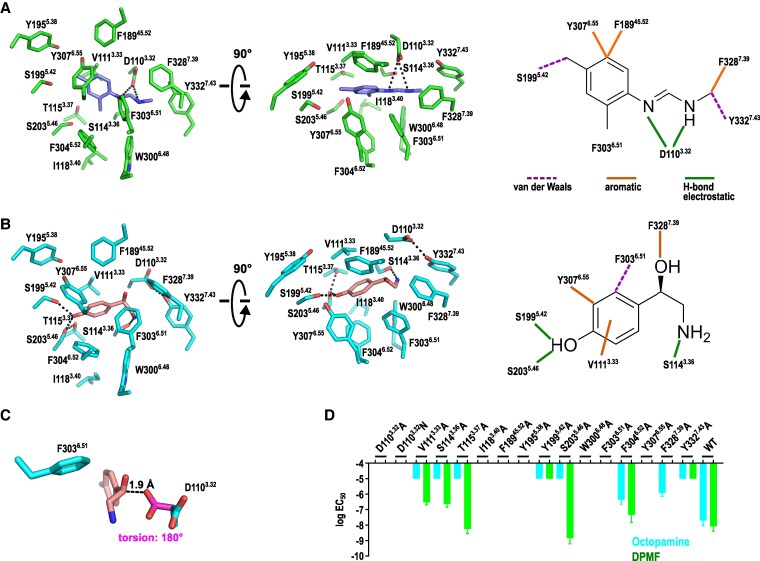
Agonist binding mode. a and b) Agonist binding mode for DPMF (A) and octopamine (B). The types of interactions are presented in Table S6. C) Structure of *I. scapularis* octβ_2_R (is-octβ_2_R)/oct at the D110^3.32^ site. For clarity, only residues D110s^3.32^ and F303^6.51^ are demonstrated. The D110^3.32^ side chain model with a 180° torsion angle about the Cα–Cβ bond is demonstrated with magenta carbons. D) Results of TGFα shedding assay for mutant is-octβ_2_Rs. Residues within 5 Å of the bound agonists are selected for the mutation study. logEC_50_ values are presented as bars. Mutants with signals at only 10^−5^ M are represented as logEC_50_ = −5. Data are presented as the mean ± standard error of the mean from three independent experiments (*n* = 3). Raw data and a summary of EC_50_ and *E*_max_ values are presented in Fig. S9 and Table S6, respectively.

The bound DPMF occupies the orthosteric binding site of is-octβ_2_R (Figs. 1C and 4A). The phenyl groups of DPMF interact with TM5, and the methylformamidine moiety is positioned near TM3, TM6, and TM7. This binding orientation aligns with the predictions from AutoDock Vina docking simulations (Fig. S8).

The distal nitrogen of the methylformamidine moiety forms hydrogen bonds with D110^3.32^ (Fig. 4A and Table S6), as observed in certain amine groups of ligands bound to various aminergic GPCRs, including β_1_ARs (44), D1R and D2R (34, 45), and α_2A_ARs (46). The methyl group of the methylformamidine moiety interacts with F328^7.39^ through CH–π interactions and with Y332^7.43^ through van der Waals interactions. The significance of these interactions is supported by the observation that 2′,4′-dimethylaniline, the other metabolite of amitraz lacking the distal nitrogen, exhibits no agonist activity at is-octβ_2_R (Figs. 1A and S2a). Additionally, the proximal nitrogen of the methylformamidine moiety formed an electrostatic interaction with D110^3.32^ (Fig. 4A). A cell-based assay using alanine mutants revealed that the Y332^7.43^A mutation significantly reduced DPMF potency, with a signal observed only at a concentration of 10^−5^ M (Figs. 4D and S9 and Table S6). Although the cell-based assay does not directly measure agonist–receptor binding, the EC_50_ values obtained serve as an indirect indicator of agonist potency under the present experimental conditions. The F328^7.39^A mutant, which disrupts the potential CH–π interaction with the methyl group, exhibited no activity. The loss of activity was not attributed to protein misfolding because the F328^7.39^A mutant retains a 10^−6^ M range of EC_50_ for octopamine. In contrast, the D110^3.32^A and D110^3.32^N mutants exhibited no activity, as observed in other aminergic GPCRs. Cell surface expressions of both D110^3.32^A and D110^3.32^N mutants were verified (Fig. S6). These findings collectively demonstrate the crucial role of the interactions between the methylformamidine moiety and D110^3.32^, F328^7.39^, and Y332^7.43^ in DPMF binding to is-octβ_2_R.

Unlike octopamine, which possesses a hydroxyl group on its phenyl ring, DPMF lacks this feature (Fig. 1A). Consequently, the dimethylphenyl moiety of the bound DPMF aromatically interacted with residues within the is-octβ_2_R binding site. Notably, the phenyl ring of F189^45.52^ at ECL2 formed edge-to-edge interactions, F303^6.51^ formed edge-to-π interactions, and Y307^6.55^ formed π–π interactions with the dimethylphenyl moiety (Table S6). Additionally, the S199^5.42^A mutation significantly reduced DPMF potency (signal observed only at 10^−5^ M) in the cell-based assay (Figs. 4D and S9 and Table S6), indicating a crucial role of S199^5.42^ in DPMF binding, likely through van der Waals interactions.

The ortho-methyl group of the dimethylphenyl moiety relative to the methylformamidine moiety is positioned near a hydrophobic pocket formed by residues S114^3.36^, L118^3.40^, W300^6.48^, F303^6.51^, and F304^6.52^ at the bottom of the orthosteric binding site (Fig. 4A). This hydrophobic interaction may determine the DPMF orientation. A 180-degree rotation of the DPMF molecule positions the ortho-methyl group away from the hydrophobic pocket, resulting in potential contacts only with F189^45.52^ and D110^3.32^. In this case, a portion of the ortho-methyl group becomes solvent-exposed, which may be energetically unfavorable. In this rotated configuration, the methylformamidine moiety faces away from D110^3.32^, F328^7.39^, and Y332^7.43^, disrupting the interactions and potentially reducing the binding potency. These observations highlight the significance of both methylformamidine and dimethylphenyl moiety interactions for high-potency DPMF binding to is-octβ_2_R.

### Octopamine binding mode to is-octβ_2_R

Bound octopamine was observed at the bottom of the orthosteric binding site in is-octβ_2_R/oct, occupying a position nearly identical to that of the bound DPMF in is-octβ_2_R/DPMF (Figs. 1E and 4B). The EM map confirmed that the 2-amino-1-hydroxyethyl moiety of bound octopamine exhibited R-form chirality, consistent with the configuration of bound noradrenaline in ARs (12–16).

Continuous cryo-EM density was observed between is-octβ_2_R and bound octopamine at residues V111^3.33^, S114^3.36^, Y307^6.55^, and F328^7.39^, indicating their involvement in octopamine binding (Fig. S10b). Unexpectedly, the D110^3.32^ side chain did not form the expected salt bridge or hydrogen bond with the 2-amino-1-hydroxyethyl moiety of the bound octopamine (Figs. 4B and C and S10b). Instead, it adopted an alternative rotamer conformation (dihedral angle of 92° about the Cα–Cβ bond) to interact with Y332^7.43^, as observed in various aminergic GPCR structures (PDB IDs: 6KUY, 7DH5, 8JSO, and 8W87). This deviates from the typical D^3.32^ rotamer conformation (∼180° about the Cα–Cβ bond) in numerous aminergic GPCRs, including is-octβ_2_R/DPMF (Fig. 4C). Consequently, the amine group of octopamine formed a hydrogen bond with the hydroxyl group of S114^3.36^. This interaction was crucial for octopamine binding, as demonstrated by the significantly reduced potency for octopamine (signal observed only at 10^−5^ M) in the S114^3.36^A mutant.

**Fig. 5.**
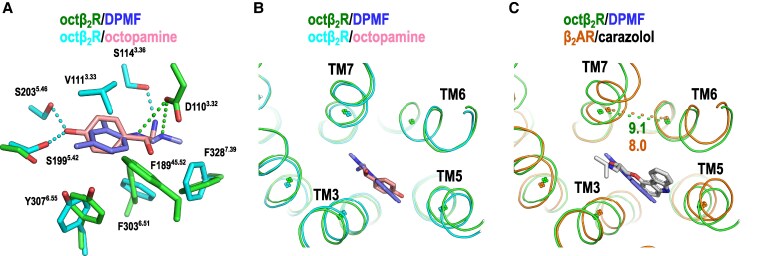
Structural comparison. A) Superposition of *I. scapularis* octβ_2_R (is-octβ_2_R)/DPMF and is-octβ_2_R/oct structures at the orthosteric binding site. Only residues associated with agonist binding are presented. The types of interactions are presented in Table S6. B and C) Superposition of is-octβ_2_R/DPMF and is-octβ_2_R/oct (B) and is-octβ_2_R/DPMF and β_2_AR/carazolol (inactive structure) (C). Distances are measured between the centers of gravity of the Cα atoms of residues 6.50–6.52 and 7.38–7.40 in TM 6 and 7, respectively. All distances related to TM 3, 5, 6, and 7 are listed in Table S7.

The 2-amino-1-hydroxyethyl moiety of the bound octopamine is sandwiched between the Cβ atom of D110^3.32^ and phenyl ring of F303^6.51^ (Fig. 4C). The conformation of F303^6.51^ differs from that of is-octβ_2_R/DPMF, which is stabilized by a series of unique edge-to-π or π–π interactions with F328^7.39^, F303^6.51^, F304^6.52^, and F204^5.47^ (Fig. 5A). Additionally, the orthosteric binding site of is-octβ_2_R/oct exhibited narrower dimensions between TM3 and TM6, TM3 and TM7, TM5 and TM7, and TM6 and TM7 than those of is-octβ_2_R/DPMF—with differences exceeding 0.5 Å (Fig. 5B and Table S7). Therefore, the putative typical rotamer conformation of the D110^3.32^ side chain (∼180° about the Cα–Cβ bond)—observed in numerous aminergic GPCRs—may result in steric clashes between the chiral carbon of the 2-amino-1-hydroxyethyl group and carboxyl oxygens of D110^3.32^ within the is-octβ_2_R/oct complex (Fig. 4C). This observation indicates that the wide range of structural contexts of is-octβ_2_R affects the rotamer conformation of D110^3.32^.

The 1-hydroxyl group of the 2-amino-1-hydroxyethyl moiety formed a π-hydrogen bond with F328^7.39^ (Fig. 4B). This interaction is crucial because the F328^7.39^A mutation significantly reduced the potency for octopamine to the 10^−6^ M range (Figs. 4D and S9, and Table S6). Additionally, tyramine, lacking the 1-hydroxyl group, exhibited weaker potency for is-octβ_2_R than octopamine (Fig. 1A), indicating that this interaction is crucial for octopamine binding.

The hydroxyl group of the phenol moiety formed hydrogen bonds with S199^5.42^ and potentially S203^5.46^ (Fig. 4B). The S199^5.42^A mutation significantly reduced the potency of octopamine (signal observed only at 10^−5^ M), supporting the hydrogen bond formation between the phenol hydroxyl group and S199^5.42^. Although the EM map did not definitively support the rotamer conformation of S203^5.46^, the S203^5.46^A mutation also resulted in a very weak octopamine potency (signal observed only at 10^−5^ M) with no effect on DPMF potency (EC_50_ = 1.3 nM) (Fig. 4D). In this putative rotamer, the S203^5.46^ hydroxyl group may form hydrogen bonds with the hydroxyl group of T115^3.37^. Consistently, the T115^3.37^A mutation significantly reduced octopamine potency (signal observed only at 10^−5^ M), while minimally affecting DPMF potency (EC_50_ = 5.0 nM). These findings indicate that hydrogen bond interactions among the bound octopamine, S199^5.42^, S203^5.46^, and T115^3.37^ are crucial for high-potency signal transduction. The residues S^5.42^ and S^5.46^, along with D^3.32^, form the D^3.32^-S^5.42^-S^5.46^ motif that is crucial for ligand binding in ARs and DRs (10, 14). Homologous interactions involving these residues are observed in other ARs and DRs (10–16).

The phenol moiety of the bound octopamine at the meta position relative to the 2-amino-1-hydroxyethyl group forms edge-to-edge interactions with Y307^6.55^ (Fig. 4B). The significance of this edge-to-edge interaction is underscored by the observation that noradrenaline and dopamine, which possess a hydroxyl group at the corresponding meta position, exhibit significantly weaker affinities for is-octβ_2_R than octopamine (Fig. 1A).

Finally, the phenol moiety of the bound octopamine may form CH–π interactions with the γ-methyl groups of V111^3.33^ (Fig. 4B). The V111^3.33^A mutation significantly reduced octopamine potency (signal observed only at 10^−5^ M) but minimally affected DPMF potency (EC_50_ = 320 nM) (Figs. 4D and S9 and Table S6). In the is-octβ_2_R/DPMF complex, the γ-methyl groups of V111^3.33^ are positioned off-center relative to the plane of the 2,4-dimethyl-phenyl group of DPMF, unlike the bound octopamine in the is-octβ_2_R/oct complex (Fig. 4A).

**Fig. 6.**
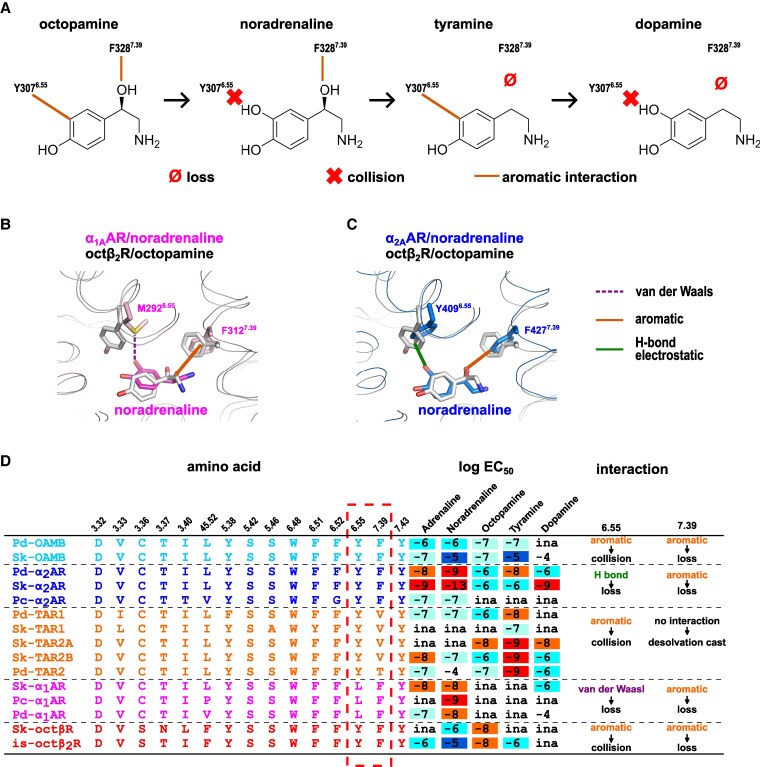
Selectivity of TDAs for aminergic receptors. (A) Schematic illustration of the putative binding mode for TDAs to *I. scapularis* octβ_2_R (is-octβ_2_R). “Loss” and “collision” indicate the disappearance of interactions and steric hindrance in the putative is-octβ_2_R and corresponding TDA complexes, respectively. B and C) Comparison of the is-octβ_2_R/oct structure with human α_1A_AR/noradrenaline (PDB ID: 7YMH) (B) and human α_2A_AR/noradrenaline (PDB ID: 7EJ0) (C). D) Amino acid conservation, a summary of assay results by Bauknecht and Jékely,^29^ and possible interactions with respective endogenous agonists and other TDAs. The values of is-octβ_2_R are from this study. As the same endogenous agonist pair, putative interactions involving residues 6.55 and 7.39 of α_1_AR, α_2_AR, and the OAMB are inferred from human α_1A_ARs, human α_2A_ARs, and is-octβ_2_R, respectively. Owing to the lack of structural data on tyramine-related receptors, putative interactions involving residue 6.55 of tyramine receptors are inferred from is-octβ_2_R, based on the identity of the functional group. The considered interactions with TDAs other than endogenous agonists are listed under the arrows.

The is-octβ_2_R/oct structure demonstrated the high selectivity of is-octβ_2_R for octopamine (Fig. 1A). This selectivity arises from a combination of specific interactions, including edge-to-edge, edge-to-π, CH–π, and π-hydrogen bonds, in addition to conventional hydrogen bonding. If other TDAs bind to is-octβ_2_R at a position similar to that of octopamine in is-octβ_2_R/oct, their lower potency can be attributed to the disruption of one or more of these specific interactions during binding (Fig. 6A). Specifically, dopamine and tyramine lack the 1-hydroxyl group in the 2-amino-1-hydroxyethyl moiety, preventing the formation of the crucial π-hydrogen bond with F328^7.39^ that is observed in octopamine. Additionally, noradrenaline and dopamine possess an additional meta-hydroxyl group on the phenol moiety that likely disrupts the edge-to-edge interaction with Y307^6.55^. These structural differences in TDAs are associated with their observed order of potency (EC_50_): octopamine > tyramine > noradrenaline > dopamine (Fig. 1A). Octopamine, with all interactions intact, exhibited the highest potency, whereas dopamine, lacking both the π-hydrogen bond with F328^7.39^ and the edge-to-edge interaction with Y307^6.55^, exhibited the lowest potency. These results indicated that Y307^6.55^ and F328^7.39^ are the primary residues that determine the TDA potency for is-octβ_2_R.

### Comparison of the binding modes between is-octβ_2_R/DPMF and is-octβ_2_R/oct, and the consideration of the activating mechanism

The overall structures of the is-octβ_2_R/DPMF and is-octβ_2_R/oct complexes were highly similar, with subtle structural differences at the orthosteric binding site (Fig. 5B). Although the residues involved in agonist binding are not completely shared between them, 4 residues—S199^5.42^, F303^6.51^, Y307^6.55^, and F328^7.39^—play crucial roles in recognizing both agonists (Figs. 4A, B and 5A and Table S6).

To elucidate the activation mechanism of is-octβ_2_R by agonists, we compared the structures of is-octβ_2_R (is-octβ_2_R/DPMF and is-octβ_2_R/oct) with the inactive state of the β_2_AR structure bound to carazolol (β_2_AR[ina]) because no inactive-state structures of octβ_2_R or D1R are currently available (Fig. 5C and Table S7). The distances between TM6 and TM7 within the orthosteric binding site were greater in both is-octβ_2_Rs compared with those in β_2_AR(ina), although the differences are not substantial, measuring 1.1 and 0.6 Å for is-octβ_2_R/DPMF and is-octβ_2_R/oct, respectively (Fig. 5C and Table S7). In contrast, the inter-TM distances involving TM3, TM5, TM6, and TM7 did not exhibit significant features, excluding those between TM6 and TM7.

The separation of TM6 and TM7 can be induced by the interaction with F303^6.51^, Y307^6.55^, and F328^7.39^, the common binding interactions of DPMF or octopamine with is-octβ_2_R (Table S6). Notably, Y307^6.55^ and F328^7.39^ are the primary residues determining the selectivity of is-octβ_2_R for octopamine among TDAs. These putative structural alterations within the orthosteric binding site will be transmitted to the G-protein binding site through conserved motifs, such as the Na^+^-binding site, PIF, NPXXY, and DRY motifs, as previously described for other class-A GPCRs (31, 33). It is important to compare the structure of the inactive state of is-octβ_2_R to fully understand its activation mechanism.

## Discussion

This study demonstrated that the homologous amitraz-resistant Y215^5.58^H mutation alters the is-octβ_2_R efficacy. Residue Y^5.58^ has been previously identified as crucial for G-protein activation in class-A GPCRs^43^. As anticipated, the Y^5.58^H mutation significantly attenuated the efficacy of is-octβ_2_R activation by DPMF, amitraz, and octopamine, resulting in an ∼50% reduction in each case. While a 50% reduction in DPMF efficacy may be insufficient for effective mite population control, a similar reduction in octopamine efficacy may not sufficiently disrupt essential physiological processes in mites.

Notably, the Y^5.58^H mutation did not significantly affect the potency of is-octβ_2_R (EC_50_) for any of the tested agonists, indicating that the mutation did not significantly alter the orthosteric binding site conformation. This indicates that although agonist binding remains unaffected, the Y^5.58^H mutation may impair the receptor's cytoplasmic region from fully activating G-protein (38–41). Consequently, enhancing the efficacy of novel orthosteric agonists in the Y^5.58^H mutant may be challenging. Although structural data of the Y^5.58^H mutant may be necessary for elucidating the molecular mechanism of its reduced efficacy, targeting allosteric sites on the cytoplasmic face of the receptor—a strategy successfully used in other GPCR systems—may offer a more promising avenue for developing novel acaricides against mites harboring the Y^5.58^H mutation (47).

This study demonstrated that two primary interactions—edge-to-edge interaction with Y307^6.55^ and π-hydrogen bond with F328^7.39^—contribute to the selectivity of is-octβ_2_R for TDAs. Both residues are involved in the activation of is-octβ_2_R, including the expanding movement between TM6 and TM7 at the orthosteric binding site (Fig. 5C and Table S7). The lower potency of other TDAs compared with octopamine for is-octβ_2_R can be attributed to the absence of one or both significant interactions with Y307^6.55^ and F328^7.39^ (Fig. 6A). With the exception of residues 6.55, 7.39, and 45.52, the orthosteric binding site of aminergic GPCRs is identical to that of is-octβ_2_R, including the homologous residue C/S^3.36^ (Fig. 6D and Tables S1 and S8). In this study, we hypothesized that the reduced affinities of TDAs, other than their endogenous agonists, for each aminergic receptor result from the absence of one or both interactions with residues 6.55 and 7.39, as discussed for is-octβ_2_R (Fig. 6A).

We describe the applicability of this hypothesis to invertebrate aminergic GPCRs, focusing on the primary residues 6.55 and 7.39. Bauknecht and Jékely (29) previously demonstrated that invertebrate homologous receptors exhibit the highest potency for the respective endogenous agonist: noradrenaline for α_1_ARs and α_2_ARs, octopamine for OAMBs and octβ_2_R, and tyramine for TARs (Figs. 1A and 6D). Additionally, numerous TDAs other than their respective agonists exhibit agonistic activity with lower potency or no signaling, similar to is-octβ_2_R (Figs. 1A and 6D) (29).

For the structure-unknown α_1_ARs, α_2_ARs, OAMB, and TARs, predicted binding interactions at residues 6.55 and 7.39 with TDAs were inferred from the structures of closely related receptors (Fig. 6D). At residue 6.55, a hydrogen bond (Y^6.55^ of α_2_AR) and van der Waals interaction (L^6.55^ of α_1_AR) may be formed with the meta-hydroxyl group of the catechol moiety of putatively bound adrenaline and dopamine (Fig. 6B and D). Although the van der Waals interaction of human α_1A_AR was not explicitly mentioned by the authors (12, 13), nuclear magnetic resonance studies of human α_1A_AR indicate that M^6.55^ contributes to adrenaline binding (48). The absence of this hydrogen bond or van der Waals interaction for the putatively bound octopamine or tyramine, which lack the meta-hydroxyl group, likely explains their reduced potency or inability to bind to α_1_ARs and α_2_ARs (Fig. 6D). Additionally, Y^6.55^ in OAMB, octβ_2_R, and TARs may form aromatic-aromatic interactions with the bound octopamine and tyramine, as observed in is-octβ_2_R/oct (Figs. 4B and 6D). Similar to is-octβ_2_R binding with noradrenaline and dopamine, a collision between Y^6.55^ and the meta-hydrogen group of the catechol moiety may result in reduced potency or loss of binding of dopamine and noradrenaline to OAMB, octβ_2_R, and TARs (Fig. 6D).

In residue 7.39, F^7.39^ of human α_1A_AR, human α_2A_AR, and is-octβ_2_R interact aromatically with the 1-hydroxyl-2-amino group of the respective bound agonists (Fig. 6A–C). Additionally, the corresponding invertebrate receptors, α_1_AR, α_2_AR, OAMB, and octβ_2_R, are expected to exhibit aromatic interactions with the residue F^7.39^ (Fig. 6D). Moreover, the reduced affinities of tyramine and dopamine for these receptors can be attributed to the absence of aromatic interactions with F^7.39^ (Fig. 6D). In contrast, residue 7.39 in TARs, vertebrate DRs, and TAAR1—lacking a hydroxyl group on the ethylamine moiety of their endogenous agonists—is typically a non-aromatic hydrophobic (predominantly V/I) or small hydrophilic (S/T) residue that does not contribute to 1-hydro-2-amine recognition in putatively bound octopamine and noradrenaline (Fig. 6D and Table S8). This indicates that the 1-hydroxy-2-amino moiety in octopamine and noradrenaline is energetically unfavorable for binding to TARs, likely because of desolvation costs.

In summary, the reduced potency of TDAs, other than their respective endogenous agonists, for each receptor can be attributed to the absence of one or both primary determinants associated with the non-conserved residues 6.55 and 7.39 (Fig. 6D). We have discussed invertebrate receptors. Based on the conservation of amino acid residues in the orthosteric binding site, we propose that this hypothesis may apply to the selectivity of vertebrate aminergic GPCRs for noradrenaline, dopamine, and trace amines (Tables S1 and S8). However, further experimental confirmation is required. While the present discussion provides a structural basis for understanding receptor activation and agonist selectivity, complementary molecular dynamics simulations that account for bound water molecules and conformational flexibility will be essential for a more quantitative and energetically grounded evaluation.

## Materials and methods

### Construction of expression vector

For is-octβ_2_R expression in *Spodoptera frugiperda* (Sf9) insect cells, the synthesized and Sf9 codon-optimized is-octβ_2_R cDNA was integrated into the pFastBac1 vector, incorporating an N-terminal hemagglutinin signal sequence, FLAG tag, tobacco etch virus (TEV) protease cleavage site, and C-terminal His_10_ tag. For the TGFα shedding assay, is-octβ_2_R cDNA was inserted into the pcDNA3.1 vector with an N-terminal 3× FLAG tag and TEV site. For is-Gαs expression, the synthesized *Escherichia coli*-optimized is-Gαs cDNA was inserted into the pCR2.1 vector, including an N-terminal N11 tag (MKDHLIHNHHKHEHAHAEH) (49, 50) and TEV site. The dominant negative mutations (S54N, G211A, E253A, D256K, K259D, R265K, T269D, I270T, and A351S) were introduced, corresponding to human dominant-negative Gαs (51). The N-terminal 1–25 residues were replaced with the N-terminal 1–18 residues of human Gαi1 for scFv16 complex formation (52). Additionally, G2A and C3S mutations were introduced to prevent unintended disulfide bond formation. This modified Gαs is referred to as is-Gαs in the main text and methods section. For human G-protein β_1_ subunit (hGβ_1_) expression, cDNA was inserted into the pFastBac1 vector with an N-terminal FLAG tag and TEV site. For human G-protein γ_2_ subunit (hGγ_2_) expression, cDNA was inserted into the pFastBac1 vector with an N-terminal His_6_ tag and TEV site. Additionally, hGγ_2_ contained a C68S mutation to prevent lipid modification in the Sf9 cells. For the cell-free synthesis of scFv16, the synthesized *E. coli*-optimized scFv16 cDNA was inserted into the pCR2.1 vector with an N-terminal N11 tag and TEV site. All mutations were introduced using standard polymerase chain reaction-based mutagenesis with the appropriate primers. To generate bacmids for the expression of is-octβ_2_R, hGβ_1_, and hGγ_2_, each vector was transformed into *E. coli* DH10Bac (Invitrogen, Waltham, MA, United States) or MultiBac (Geneva Biotech, Geneva, Switzerland).

### Expression and purification of is-octβ_2_R

Recombinant baculovirus was produced using the Bac-to-Bac Baculovirus Expression System (Invitrogen). Sf9 cells were cultured to a density of 2.0 × 10^6^ cells/mL and infected with the recombinant baculovirus at a multiplicity of infection of 0.5. After 48 h post-infection, the cells were harvested and stored at −80 °C.

For is-octβ_2_R purification, Sf9 cells were lysed using a hypertonic buffer (50 mM Tris-Cl [pH 8.0], 800 mM NaCl, and 5% glycerol), followed by ultracentrifugation (100,000 × *g*, 4 °C, 1 h). The resulting membrane pellet was suspended in buffer A (20 mM 2-(4-(2-hydroxyethyl)piperazin-1-yl)ethanesulfonic acid [HEPES] [pH 7.5], 100 mM NaCl, and 5% glycerol), homogenized using a Potter homogenizer, and incubated with 100 μM DPMF (FUJIFILM Wako Pure Chemical Corporation, Osaka, Japan) or octopamine (Tokyo Chemical Industry Co., Ltd., Tokyo, Japan) for 3 h at 4 °C to enable agonist binding. Solubilization was achieved by adding one-tenth the volume of buffer A containing 10% (w/v) lauryl maltose neopentyl glycol (LMNG) and 1% (w/v) cholesteryl hemi-succinate (CHS). After a 2 h incubation at 4 °C, the solution was ultracentrifuged (100,000 × *g*, 4 °C, 30 min), and the supernatant was incubated with TALON resin (Clontech, Mountain View, CA, United States) overnight at 4 °C. Subsequently, the resin was packed into a gravity-flow column and washed with 20 column volumes (CV) of buffer B (buffer A containing 0.02% [w/v] LMNG, 0.002% [w/v] CHS, and 100 μM agonist). is-octβ_2_R was eluted with five CV of TALON elution buffer 1 (buffer B containing 100 mM imidazole [pH 8.0]). The eluted fraction was subsequently bound to M2 anti-FLAG resin (Sigma–Aldrich, St. Louis, MO, United States) for 2 h at 4 °C. Following another gravity-flow column packing and a 20 CV wash with buffer B, is-octβ_2_R was eluted with 5 CV of FLAG elution buffer (buffer B containing 3× FLAG peptide). The eluted fraction was concentrated using an Amicon Ultra filter (100 kDa MWCO; Millipore, Burlington, MA, United States) and further purified by gel filtration on a Superose-6 column. For cryo-EM sample preparation, the purified is-octβ_2_R was concentrated to ∼100 μL and used for complex formation with G-protein and scFv16, as described below.

### Expression and purification of *I. scapularis* G-protein α-subunit

is-Gαs was expressed in *E. coli* KRX cells. A single transformed colony was inoculated into Luria–Bertani (LB) medium containing 100 μg/mL ampicillin and cultured overnight at 37 °C. Subsequently, this pre-culture was used to inoculate 1 L of LB medium supplemented with 100 μg/mL ampicillin, 0.15% glucose, and 0.1% rhamnose, followed by further incubation at 25 °C overnight. The *E. coli* cells were harvested by centrifugation (2,350 × *g*, 30 min) and stored at −30 °C.

The stored *E. coli* cells were thawed and suspended in buffer C (50 mM Tris [pH 8.0], 300 mM NaCl, 1 mM MgCl_2_, and 50 μM GDP). The *E. coli* cells were lysed by sonication, and the lysate was centrifuged (100,000 × *g*, 15 min). The resulting supernatant was loaded onto a HisTrap column (Cytiva, Marlborough, MA, United States) pre-equilibrated with buffer C, and the bound is-Gαs was washed with buffer C. is-Gαs was eluted using a linear gradient of buffer C and HisTrap-B1 buffer (buffer C containing 500 mM imidazole [pH 8.0]). In-house TEV protease solution was added to the eluate, and the mixture was incubated at 4 °C overnight and dialyzed against buffer C to remove imidazole. Subsequently, the TEV protease-treated solution was passed through a Ni-sepharose column by gravity flow to remove His-tagged TEV protease and any contaminant proteins. The unbound fraction was further purified by gel filtration using a Superdex200 column (Cytiva) equilibrated with buffer C. Finally, the purified is-Gαs was aliquoted, flash-frozen in liquid nitrogen, and stored at −80 °C.

### Expression and purification of human G-protein βγ-subunits

hGβ1 and hGγ2 were co-expressed using the Sf9-baculovirus system, as described above. For hGβγ purification, Sf9 cells were lysed in a hypotonic buffer (50 mM Tris-Cl [pH 8.0], 50 mM NaCl, and 5% glycerol), and the lysate was ultracentrifuged (100,000 × *g*, 4 °C, 1 h). The resulting supernatant was incubated with TALON resin for 2 h at 4 °C. Subsequently, the resin was packed into a gravity-flow column, washed with 20 CV of buffer C, and eluted with 5 CV of TALON elution buffer 2 (50 mM Tris [pH 8.0], 300 mM NaCl, and 100 mM imidazole [pH 8.0]). The TEV protease solution was added to the eluate, and the mixture was incubated overnight at 4 °C and dialyzed against buffer D (50 mM Tris [pH 8.0] and 300 mM NaCl). The TEV protease-treated solution was subsequently passed through a Ni-sepharose column through gravity flow to remove the His-tagged TEV protease and any contaminating proteins. The unbound fraction was further purified by gel filtration using a Superdex200 column (Cytiva) equilibrated with buffer D. Finally, the purified hGβγ complex was aliquoted, flash-frozen in liquid nitrogen, and stored at −80 °C.

### Preparation of trimeric G-protein

For trimeric G-protein preparation, excess is-Gαs and human Gβγ were mixed and incubated overnight at 4 °C. The resulting mixture was purified by gel filtration using a Superdex 200 column equilibrated with buffer A. Subsequently, fractions containing the heterotrimeric complex were used for complex formation with is-octβ_2_R and scFv16.

### Preparation of scFv16

scFv16 was prepared using an *E. coli* cell-free protein synthesis system (53, 54). To facilitate proper disulfide bond formation, 0.4 mg/mL disulfide bond isomerase C protein was added to the reaction solution, and 5 mM glutathione disulfide and 0.75 mM dithiothreitol were included in both the reaction and feeding buffers. scFv16 was synthesized overnight at 15 °C using 2 μg/mL of plasmid. Subsequently, the reaction solution was centrifuged (100,000 × *g*, 15 min). The resulting supernatant was purified using a HisTrap column with buffers C and HisTrap-B1. TEV protease solution was added to the eluate, and the mixture was incubated overnight at 4 °C while being dialyzed against buffer D to remove imidazole. The TEV protease-treated solution was subsequently passed through a Ni-sepharose column through gravity flow to remove the His-tagged TEV protease and contaminant proteins. The unbound fraction was further purified by gel filtration using a Superdex200 column equilibrated with buffer A. Subsequently, the purified scFv16 was aliquoted, flash-frozen in liquid nitrogen, and stored at −80 °C.

### is-octβ_2_R, G-protein, and scFv16 complex preparation for cryo-EM measurement

The purified is-octβ_2_R, trimeric G-protein, scFv16, 10 mM MgCl_2_, 5 mM CaCl_2_, and 500 mU/mL apyrase were mixed and incubated overnight at 4 °C. An excess molar ratio of G-protein and scFv16 was used relative to is-octβ_2_R. The complex was purified by gel filtration using a Superdex200 column equilibrated with buffer E (20 mM HEPES [pH 7.5], 100 mM NaCl, 0.00075% LMNG, 0.000075% CHS, 0.00025% glyco-diosgenin, and 100 μM agonist) and concentrated to 3.0 mg/mL (DPMF complex) and 1.6 mg/mL (octopamine complex) (Fig. S11).

### Grid preparation for cryo-EM measurement and data collection

The concentrated samples were filtered through a 0.2 μm filter (Millipore). The sample (3 µL) was applied to glow-discharged, holey carbon grids (Quantifoil R0.6/1, 300 mesh, copper), blotted for 3 s at 4 °C and 100% humidity, and plunge-frozen in liquid ethane using a Vitrobot Mark IV (Thermo Fisher Scientific, Waltham, MA, United States). The grids were observed to confirm the freeze condition using a Tecnai Arctica transmission electron microscope. Cryo-EM imaging of the complexes was performed on a 300 kV Titan Krios G4 equipped with a BioQuantum K3 direct electron detector in the electron counting mode at ×105,000 magnification, corresponding to a calibrated pixel size of 0.8295 Å per pixel (Figs. S12 and S13). A total of 6,001 micrographic movies for the is-octβ2R/DPMF complex were captured using a total dose of 50.1 e^−^/Å^2^ for 48 frames with an exposure time of 2.4 s. A total of 10,622 movies for the is-octβ2R/oct complex were captured using a total dose of 50.3 e^−^/Å^2^ for 48 frames with an exposure time of 2.2 s. All data were automatically acquired using the EPU software (ver. 3.7.0.6930), with a defocus range of −0.8 to −2.0 μm.

### Cryo-EM data processing

Cryo-EM data processing was performed using cryoSPARC (ver. 4.5.3) (55) (Figs. S12 and S13). All dose-fractionated movies were subjected to beam-induced motion correction using the patch motion correction, and the patch contrast transfer function values were estimated using the default settings. Particles of the complexes were automatically picked using a blob picker with a 120–150 Å diameter blob, extracted with a box of 384 pixels, and binned to 128 pixels. Initial 3D maps were generated using ab initio 3D reconstruction. After purification through heterogeneous refinements and/or 2D classifications, the particles were re-extracted with a box of 256 pixels. For the is-octβ2R/DPMF complex, 246,183 selected particles underwent non-uniform refinement, resulting in a 3.39 Å resolution map. For the is-octβ2R/oct complex, 410,720 particles underwent non-uniform refinement, resulting in a 3.65 Å resolution map. Map enhancement with half maps and sharpening was performed using Resolve CryoEM and Auto sharpen within Phenix 1.20.1-4487 (56), respectively (Figs. S14 and S15).

### Model building and refinement

Each subunit of is-octβ_2_R/DPMF, is-Gs, and scFv16 complexes was individually rigid-body fitted in the cryo-EM map using Chimera 1.7.13 with the AlphaFold model of is-octβ_2_R (ID: A0A6P7VYN1) and Gs and scFv16 structures (PDB ID: 8JLN). The initial model of is-octβ_2_R/oct was a refined is-octβ_2_R/DPMF structure. The DPMF structure was generated from SMILE using the SwissDock site (https://www.swissdock.ch/), and the octopamine structure was obtained from the Cambridge Structure Database (ID: 1225599). Iterative manual real-space refinements were performed using Coot 0.9.8.7, followed by real-space refinement in Phenix (Figs. S10 and S14–S16). Model statistics were confirmed using MolProbity (57).

### Docking of DPMF to is-octβ_2_R

Docking calculations were performed using the online version of AutoDock Vina (58, 59). For DPMF, hydrogen atoms were added only to the distal nitrogen atom. A grid box encompassing the orthosteric binding site of is-octβ_2_R was defined to constrain the docking pose.

### TGFα shedding assay

The TGFα shedding assay was performed as previously described (28). Notably, 2 × 10^5^ HEK293 cells were seeded in 10 or 6 cm 12-well dishes for 24 h before transfection. The cells were transfected using lipofectamine 2000 with plasmids encoding alkaline phosphatase–tagged TGFα (AP-TGFα) (2.5 ng/mL), is-octβ_2_Rs (1 ng/mL), and Gαq/s (0.5 ng/mL). After 24 h, the cells were washed with phosphate-buffered saline and resuspended in Hank's balanced salt solution (HBSS) (supplemented with 5 mM HEPES [pH 7.4]). The cells (90 μL/well) were plated in a 96-well plate and incubated with 10 μL of HBSS solution containing various concentrations of compounds for 1 h at 37 °C. Following centrifugation (200 × *g*, 2 min), 80 μL of the supernatant was transferred to a separate 96-well plate. Subsequently, 80 µL of 2× para-nitrophenyl phosphate (pNPP) buffer (containing 10 mM pNPP in 40 mM Tris–HCl [pH 9.5], 40 mM NaCl, and 10 mM MgCl_2_) was added to both the supernatant and cell-containing plates. The optical density at 405 nm of both plates was measured before and after a 1 h incubation at room temperature using a microplate reader (SpectraMax iD3, Molecular Devices, San Jose, CA, United States). AP-TGFα release was calculated using the following formulas:


$$\begin{array}{l} \Delta OD_{405}=OD_{450}(1\;h)-OD_{405}(0\;h)\\ \mathrm{AP}-\mathrm{TGF}\alpha \;\mathrm{in}\;\mathrm{supernatant}\;(\text{\%})\\ =(\Delta OD_{405}\mathrm{Sup}/[\Delta \mathrm{OD}450\mathrm{Sup}+\Delta OD_{405}\mathrm{Cell}])\times 125\\ \mathrm{AP}-\mathrm{TGF}\alpha \;\mathrm{release}\;(\text{\%})\\ =\mathrm{AP}-\mathrm{TGF}\alpha \;\mathrm{in}\;\mathrm{supernatant}\;\mathrm{under}\;a\;\mathrm{stimulated}\;\mathrm{condition}\\ (\text{\%})-\mathrm{AP}-\mathrm{TGF}\alpha \;\mathrm{in}\;\mathrm{supernatant}\;\mathrm{under}\;a\;\mathrm{vehicle}-\mathrm{treated}\;\mathrm{condition} \end{array}$$

where OD_405_ (0 h) and OD_450_ (1 h) represent the OD_405_ before and after a 1 h incubation, respectively, and OD_405_Sup and OD_405_Cell represent the OD_405_ of supernatant and cell plates, respectively. For all pharmacological analyses, nonlinear regression curve fitting was performed with a 3-parameter sigmoid model (Hill coefficient = 1) using Prism 8 (GraphPad Software, La Jolla, CA, United States).

### Confocal immunofluorescence microscopy

Plasmids encoding AP-TGFα, is-octβ_2_R mutants, and Gαq/s were transfected using the same method as in the TGFα shedding assay described above. After 24 h of culture, an anti-FLAG tag antibody (Sigma–Aldrich) was added as the primary antibody at a dilution of 1:300 of the culture medium volume and incubated at 37 °C for 1 h. Cells were washed twice with Dulbecco's phosphate-buffered saline (DPBS). Next, an anti-mouse IgG antibody labeled with Alexa Fluor 488 was added as a secondary antibody at a dilution of 1:200 and incubated for an additional hour at 37 °C. Cells were stained for 10 min at 37 °C with Hoechst 33258 (Nacalai Tesque, Kyoto, Japan) and PlasMem Bright Red to label the nucleus and plasma membrane, respectively. Cells were then observed using a confocal microscope (BC43 SR, Oxford Instruments, Oxfordshire, United Kingdom).

## Supplementary Material

pgaf376_Supplementary_Data

## Data Availability

Atomic structures have been deposited at the PDB under accession numbers 9M55 (is-octβ_2_R/DPMF) https://doi.org/10.2210/pdb9M55/pdb and 9M57 (is-octβ_2_R/oct) https://doi.org/10.2210/pdb9M57/pdb The cryo-EM models and maps have been deposited at the Electron Microscopy Data Bank under accession numbers EMD-63636 (is-octβ_2_R/DPMF) doi:10.6019/EMD-63636; https://www.ebi.ac.uk/emdb/EMD-63636 and EMD-63638 (is-octβ_2_R/oct) doi:10.6019/EMD-63638; https://www.ebi.ac.uk/emdb/EMD-63638. Materials used in this study are available upon request.
