# Supplementary material for: Structural insights into endogenous agonist selectivity of aminergic receptors from the octopamine β2 receptor
Source: PNAS Nexus. 2025 Nov 28;4(12):pgaf376. doi: 10.1093/pnasnexus/pgaf376 (PMC12696353; doi:10.1093/pnasnexus/pgaf376)
Supplement: pgaf376_Supplementary_Data [file pgaf376_supplementary_data.pdf]

Supplementary Information for

Structural insights into endogenous agonist selectivity of aminergic receptors from the octopamine β2 receptor

Tetsuya Hori[a*], Kazushige Katsura[a,b], Sayako Miyamoto-Kohno[b], Tomomi Uchikubo-Kamo[a,b], Mayumi Yonemochi[b], and Mikako Shirouzu[a,b,c]

[a]Laboratory for Protein Functional and Structural Biology, RIKEN Center for Integrative Medical Sciences, 1-7-22 Suehiro-cho, Tsurumi-ku, Yokohama, Kanagawa 230-0045, Japan

[b]Drug Discovery Structural Biology Platform Unit, RIKEN Center for Integrative Medical Sciences, 1-7-22 Suehiro-cho, Tsurumi-ku, Yokohama, Kanagawa 230-0045, Japan

[c]Structural Life Science and Cell Biology Collaboration Team, RIKEN Center for Biosystems Dynamics Research, 1-7-22 Suehiro-cho, Tsurumi-ku, Yokohama, Kanagawa 230-0045, Japan

*Corresponding author

Tetsuya Hori

Email: Tetsuya.hori@riken.jp

This PDF file includes:

Supplementary text
Figures S1 to S16
Tables S1 to S8

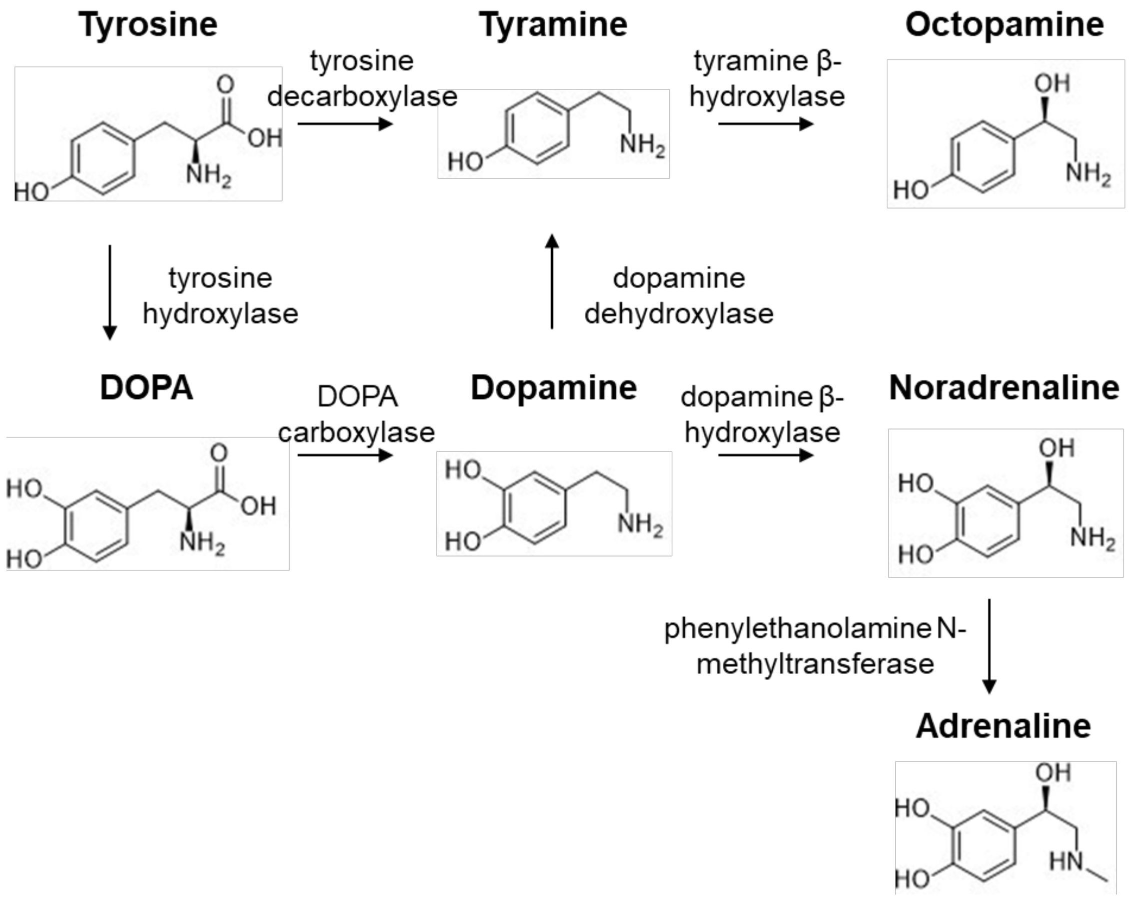

Fig. S1. Metabolite pathway of endogenous agonists from tyrosine to octopamine or adrenaline.

a

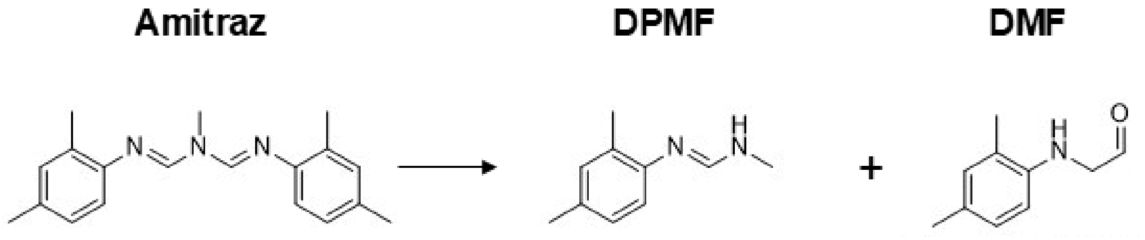

b

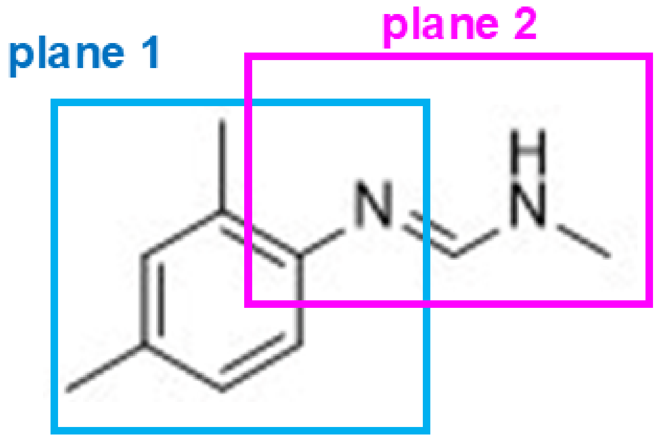

Fig. S2. Amitraz and 2,4-dimethylphenylformamide (DPMF). (a) Degradation of amitraz to DPMF and 4,4'-dimethylformamidine. (b) Chemical structure of DPMF. Protonation of the lone pair on the proximal nitrogen of the methylformamidine moiety is not favored.

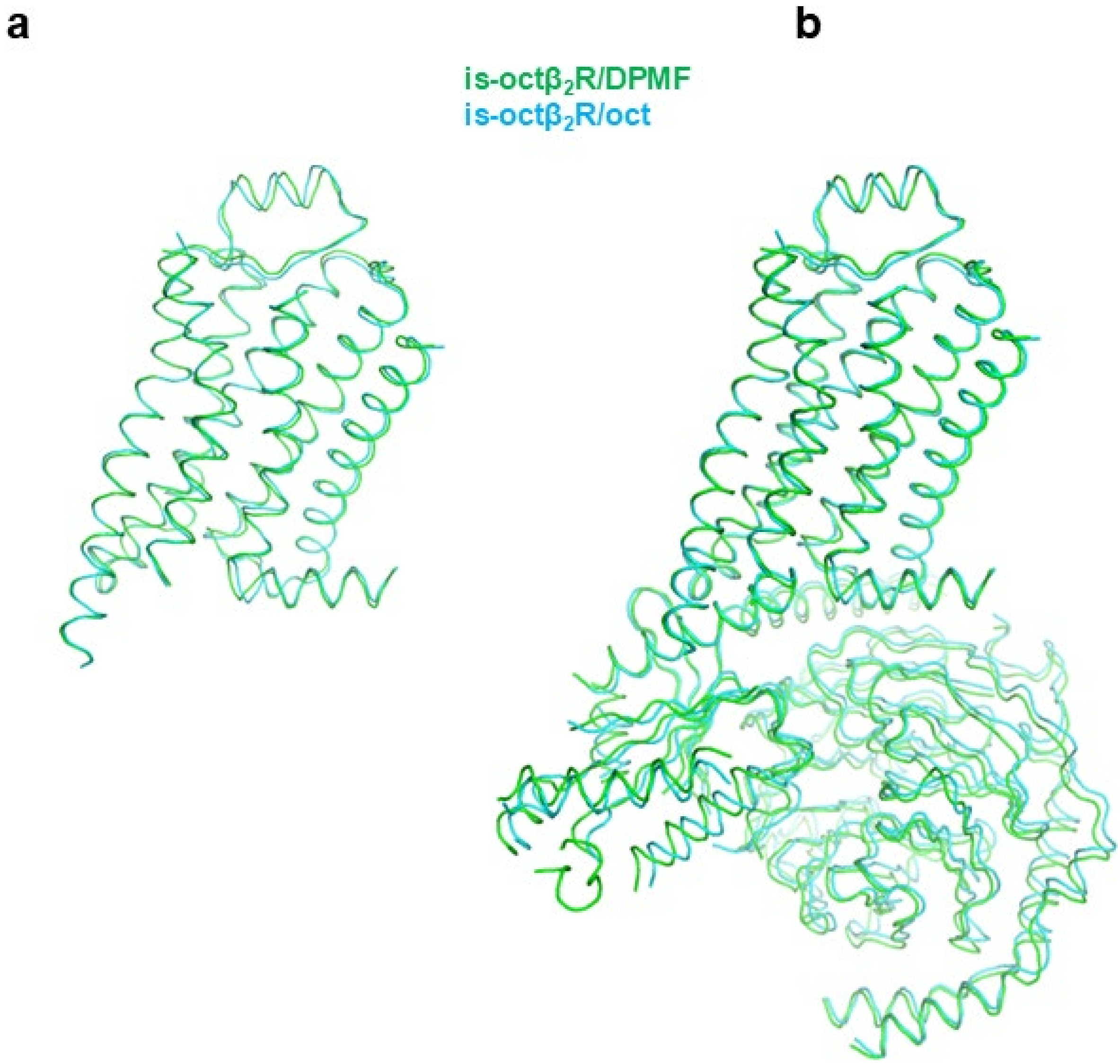

Fig. S3. Structural comparison between *I. scapularis* octβ$_2$R (is-octβ$_2$R)/2,4-dimethylphenylformamide and is-octβ$_2$R/oct. (a) is-octβ$_2$R (b) is-octβ$_2$R and Gs complex.

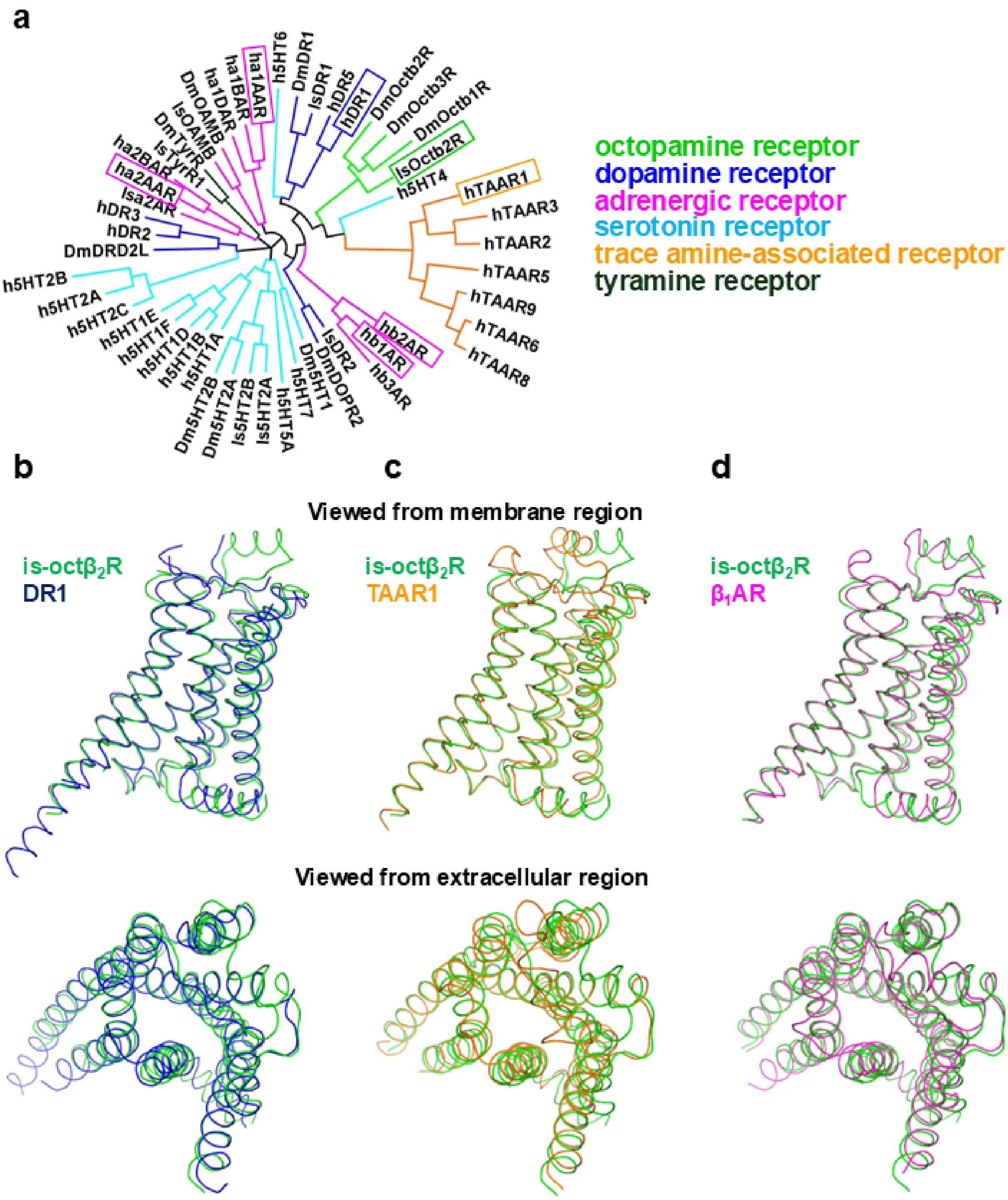

Fig. S4. Phylogenetic tree of amine G-protein coupled receptors (GPCRs) and structural comparison to *I. scapularis* octβ_2R (is-octβ_2R). (a) The phylogenetic tree includes GPCRs from *Ixodes scapularis* (prefix: is), *Drosophila melanogaster* (prefix: Dm), and humans (prefix: h), specifically focusing on receptors for octopamine, dopamine, adrenaline, serotonin, trace amine, and tyramine that are closely related to is-octβ_2R. Receptors discussed in detail in this study are highlighted with boxes. The phylogenetic tree is generated using FastTree in CLUSTALW. (b-d) Structural comparison of is-octβ_2R/DPMF with aminergic GPCRs: DR1/SKF83959 (PDB ID: 7JVP) (b), turkey β_1AR (7JJO) (c), and TAAR1 (8WC8) (d). These structures are selected based on DALI searches.

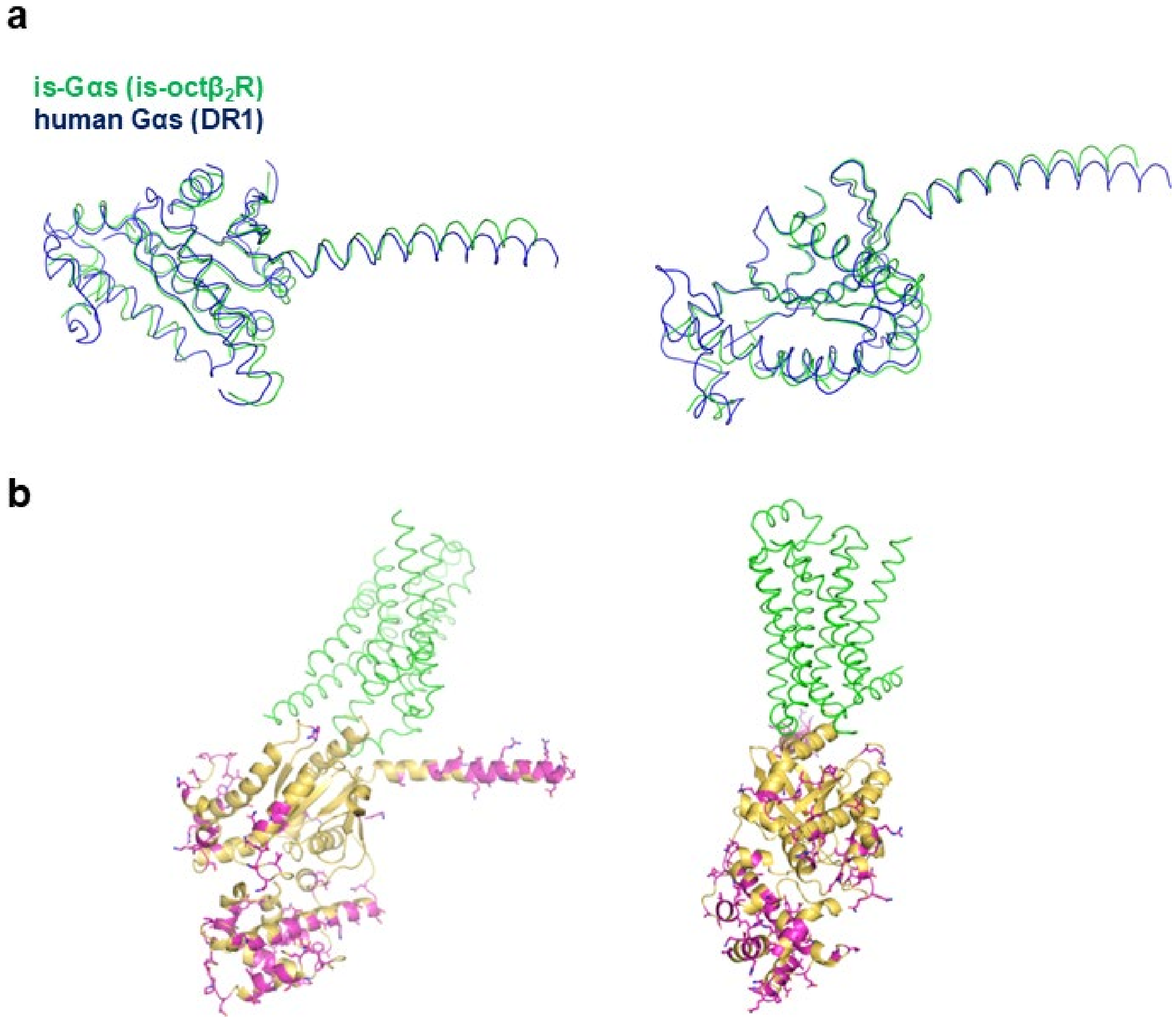

Fig. S5. About is-Gαs. (a) Structural comparison between human G-protein α subunit (Gαs) (PDB: 7JVP) and *I. scapularis* (is)-Gαs. (b) Structural mapping of residues differing between human and is-Gαs, demonstrated in magenta.

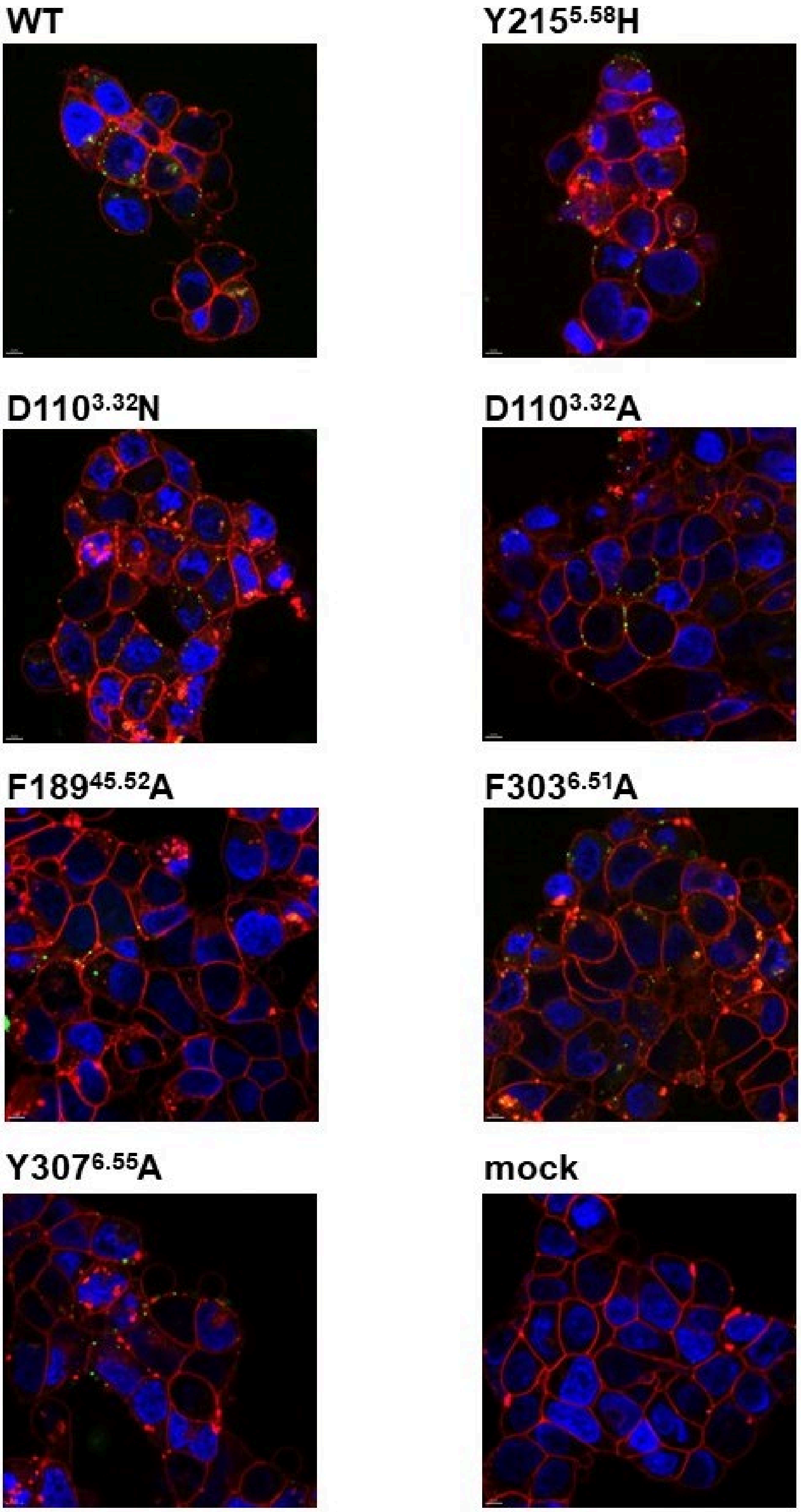

Fig. S6. Confirmation of the expression of *I. scapularis* octβ_2R mutants on the HEK293 cell surface. Cell surface expression was examined for mutants involved in DPMF or octopamine binding, which showed no signal in Fig. 4d, as well as for $Y215^{5.58}H$, which exhibited a reduced Emax in Fig. 3a.

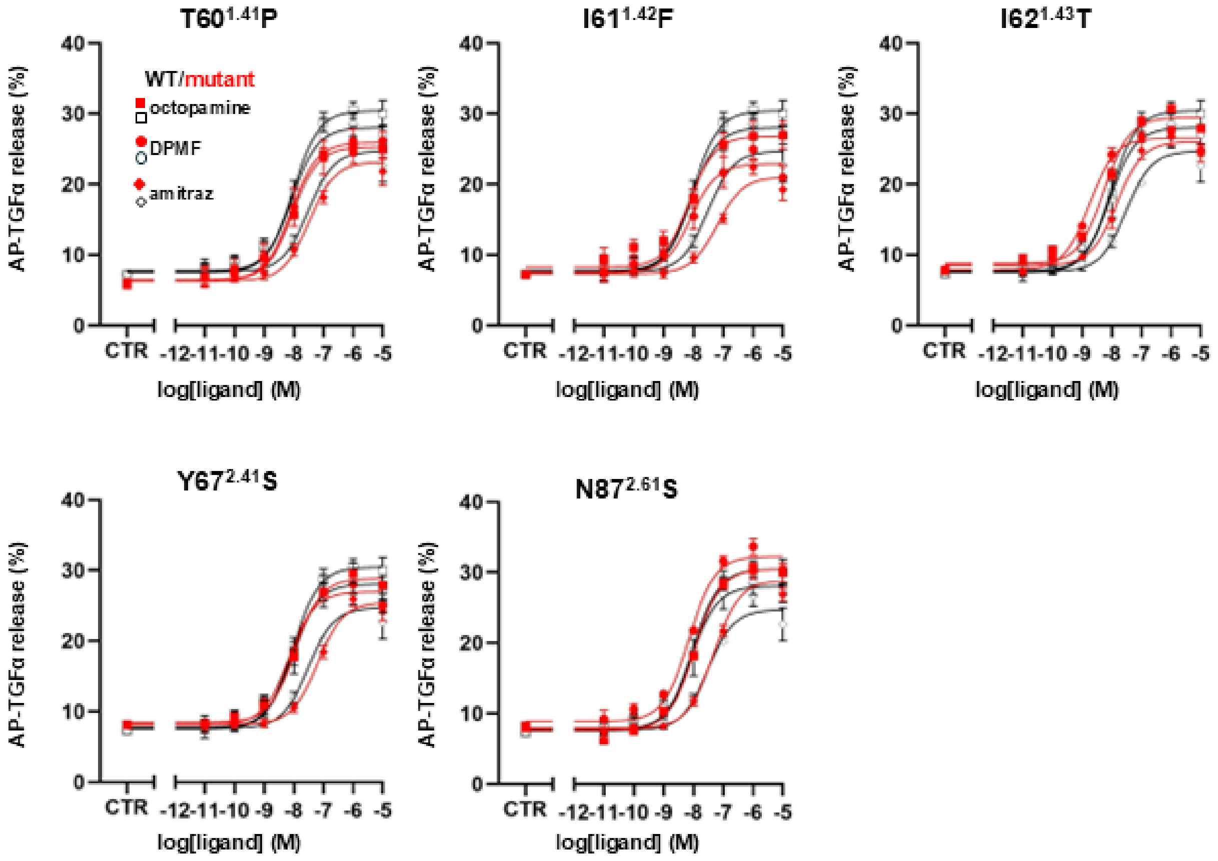

Fig. S7. Amitraz-resistant mutations. Concentration-response curves of endogenous octopamine, 2,4-dimethylphenylformamide, and amitraz for *I. scapularis* octβ$_2$R mutants, as measured by a tumor growth factor-alpha shedding assay. Data are presented as the mean ± standard error of the mean from three independent experiments (n = 3). Results for Y215$^{5.58}$H and EC_{50} and E_{max} values for all are presented in SI Appendix, Fig. 3A and Table S4.

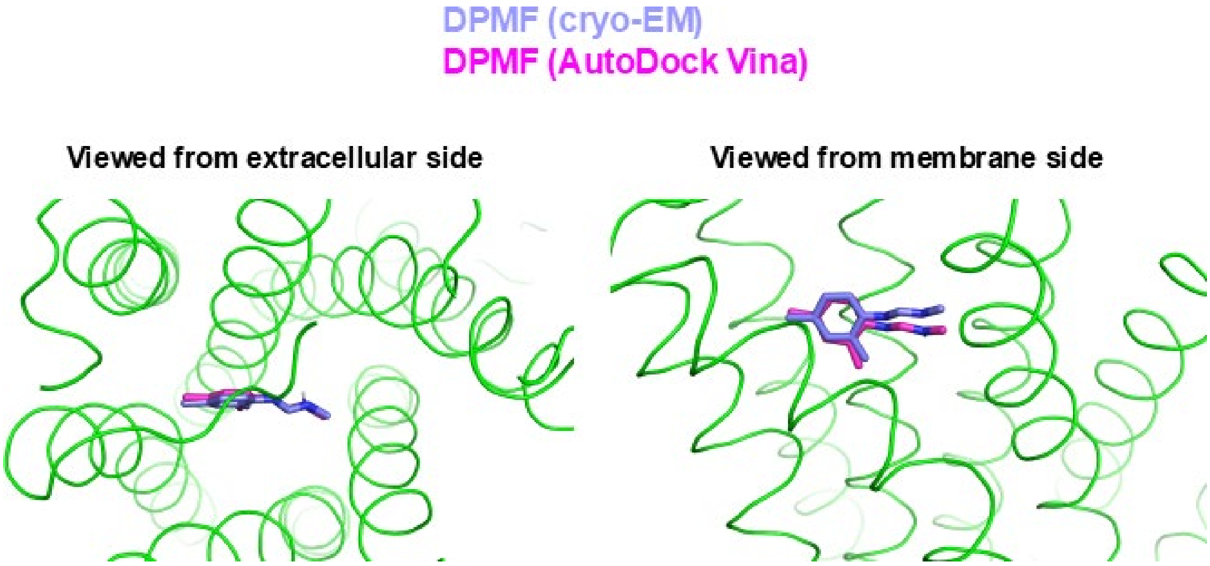

Fig. S8. Docking results of 2,4-dimethylphenylformamide (DPMF) to the *I. scapularis*-octβ_2R/DPMF structure.

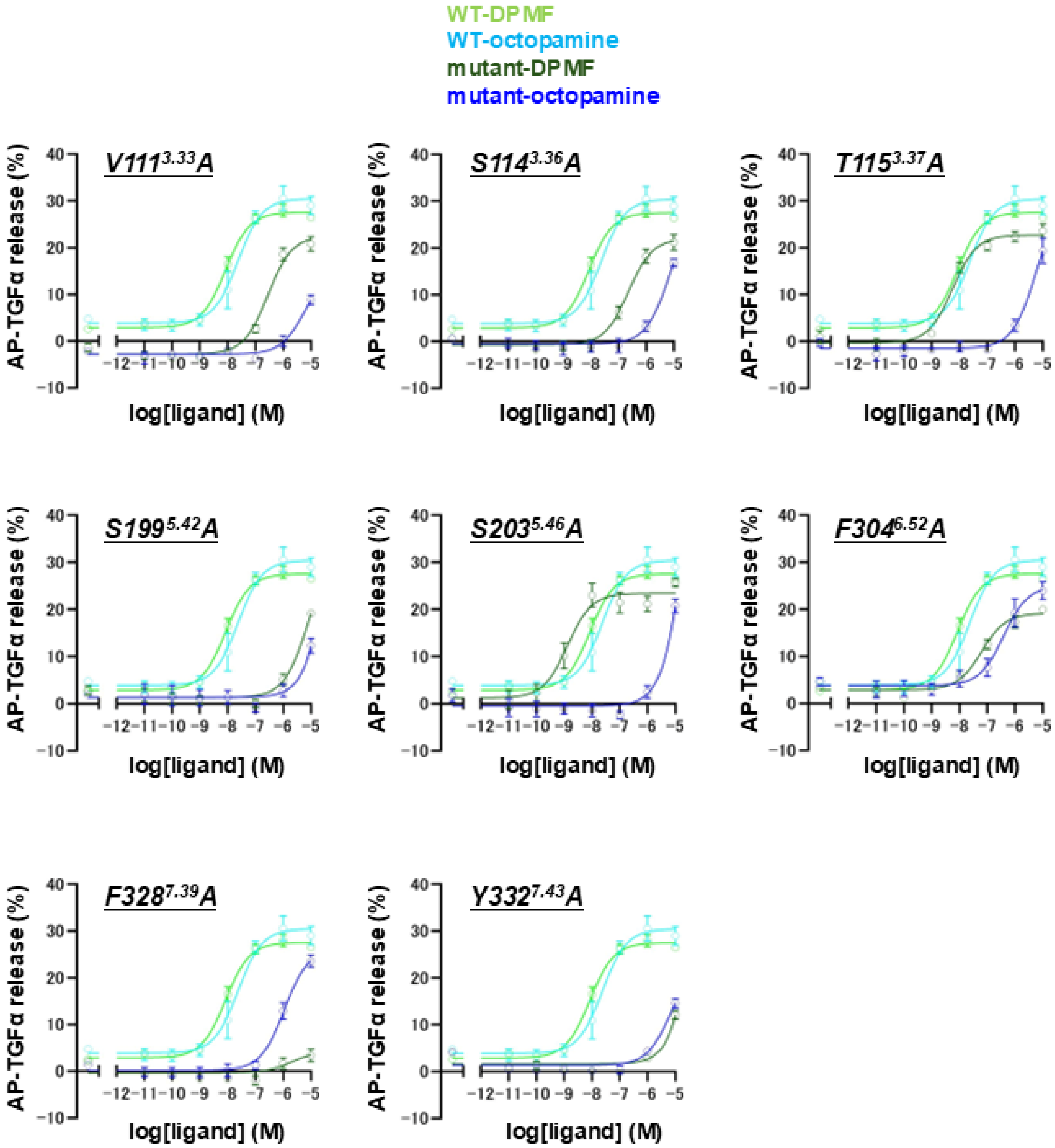

Fig. S9. Raw data from assays of *I. scapularis* (is)-octβ_2R mutants assessing agonist binding. Concentration-response curves for endogenous octopamine and 2,4-dimethylphenylformamide with is-octβ_2R mutants, as measured by a tumor growth factor-alpha shedding assay. Data are presented as the mean ± standard error of the mean from three independent experiments (n = 3). Results for wild-type and each mutant are overlaid. Only data exhibiting signals are demonstrated. A summary of logEC_{50} values is presented in Fig. 4d, and EC_{50} and E_{max} value parameters are listed in Supplementary Table S4.

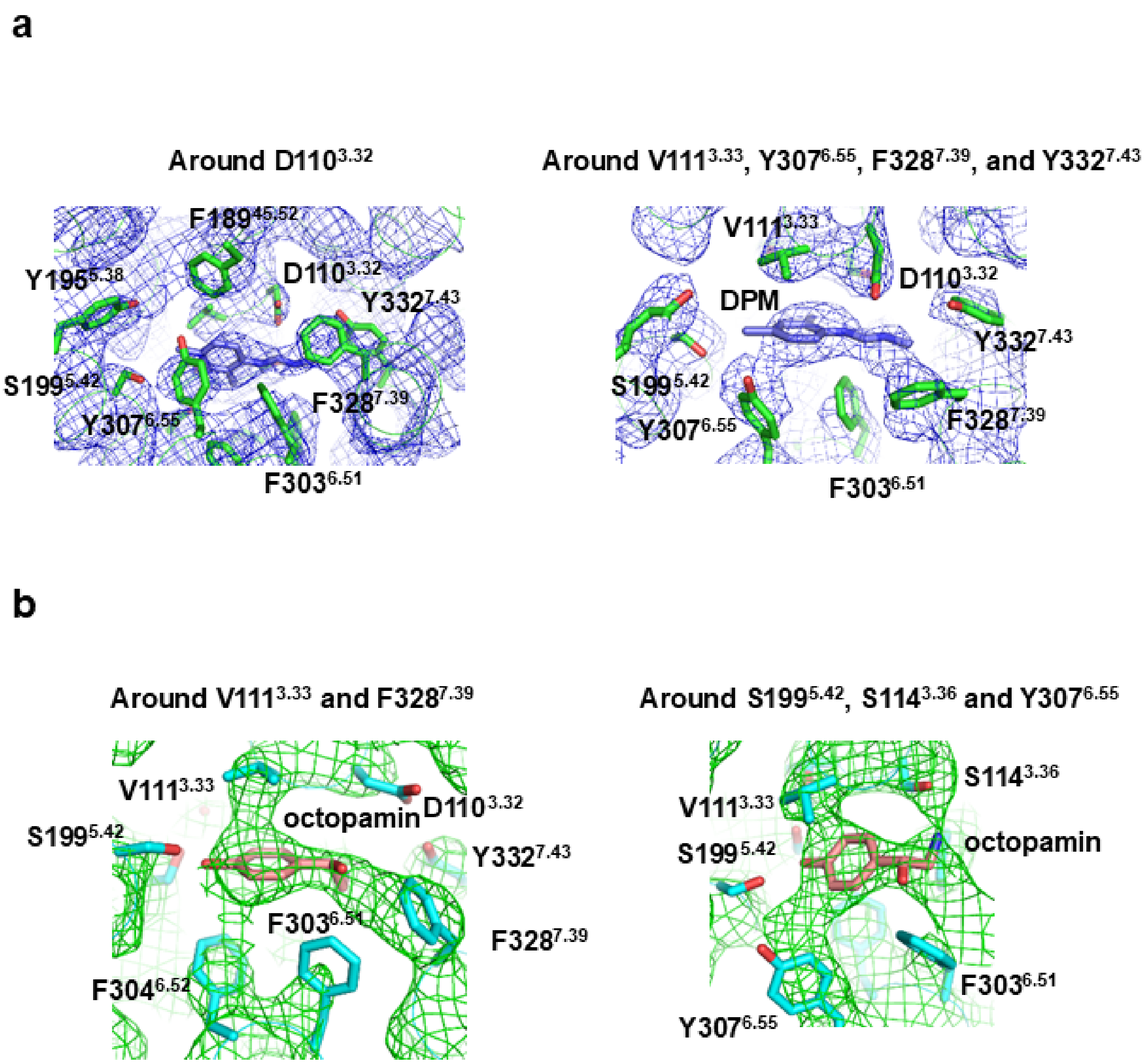

Fig. S10. Electron microscopy map about octopamine and 2,4-dimethylphenylformamide (DPMF) binding site. (a) Octopamine binding site. (b) DPMF binding site.

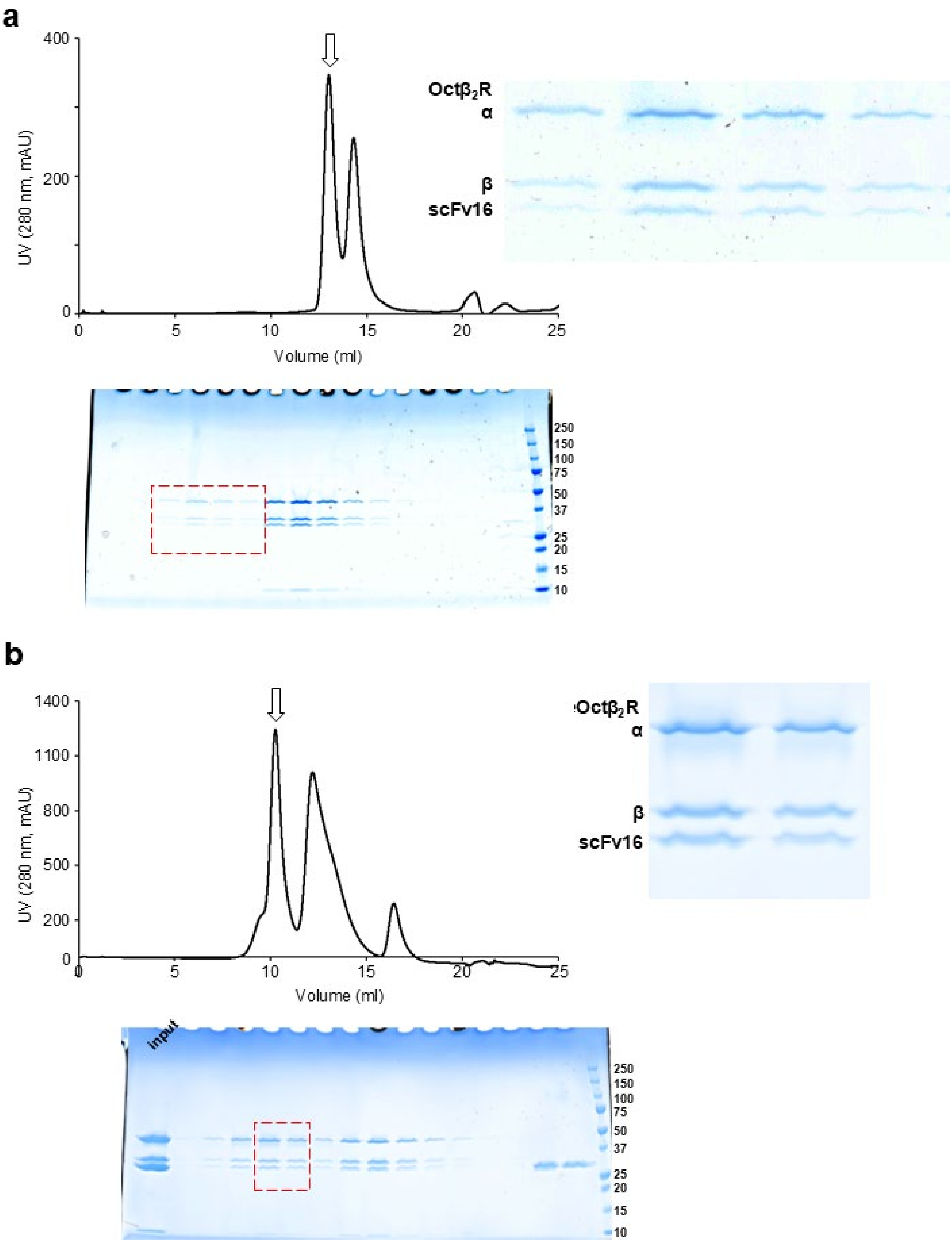

Fig. S11. Cryo-electron microscopy sample preparation. (a,b) Results for *I. scapularis* octβ_2R (is-octβ_2R)/2,4-dimethylphenylformamide (a) and is-octβ_2R/oct (b).

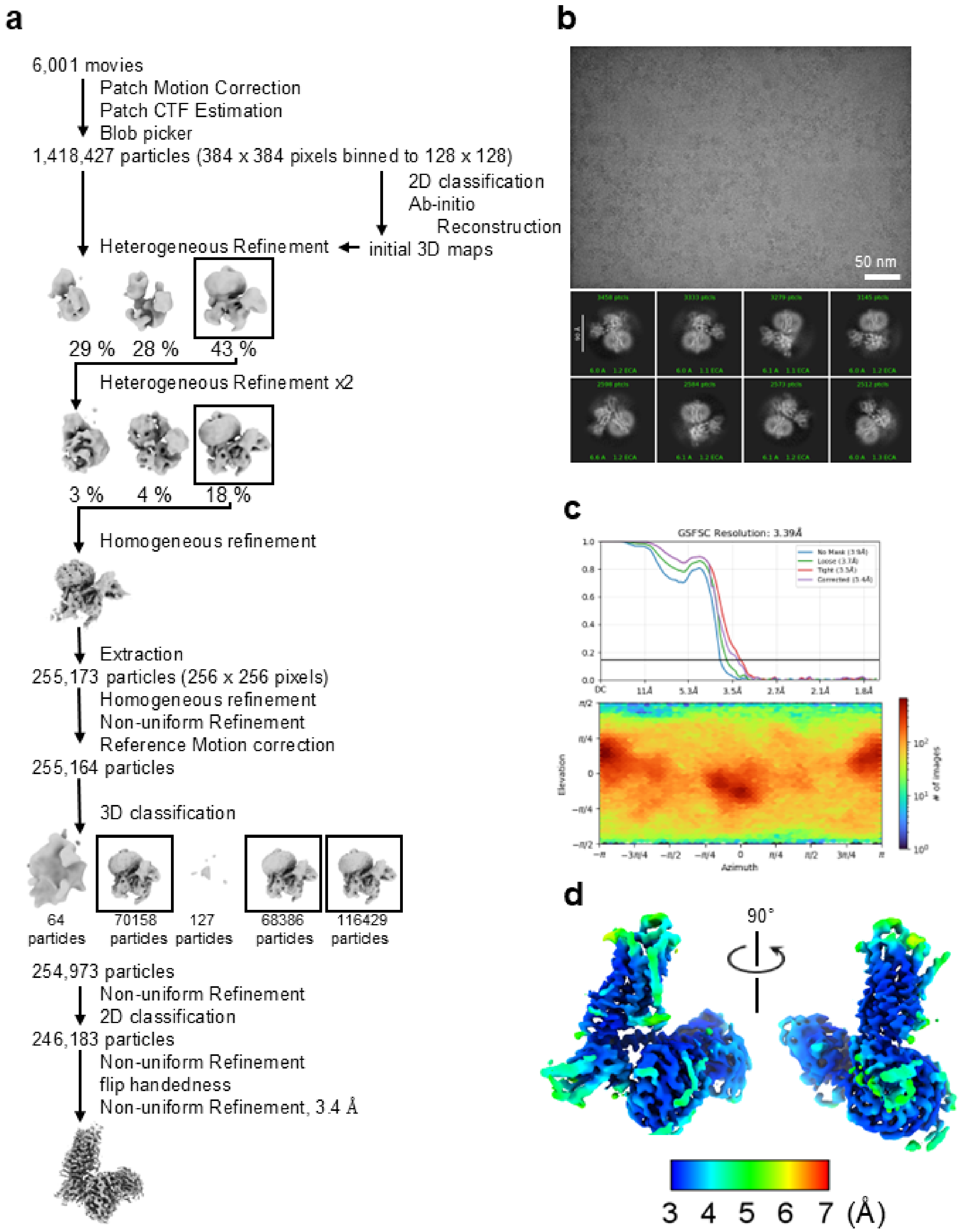

Fig. S12. Cryo-electron microscopy data processing of is-octβ$_2$R/2,4-dimethylphenylformamide. (a) Flowchart of data processing. (b) Representative micrograph and two-dimensional (2D) class averages. (c) Fourier shell correlation (FSC) curve and Euler angle distribution plot for the final refinement. (d) Final 3D density map colored by local resolution.

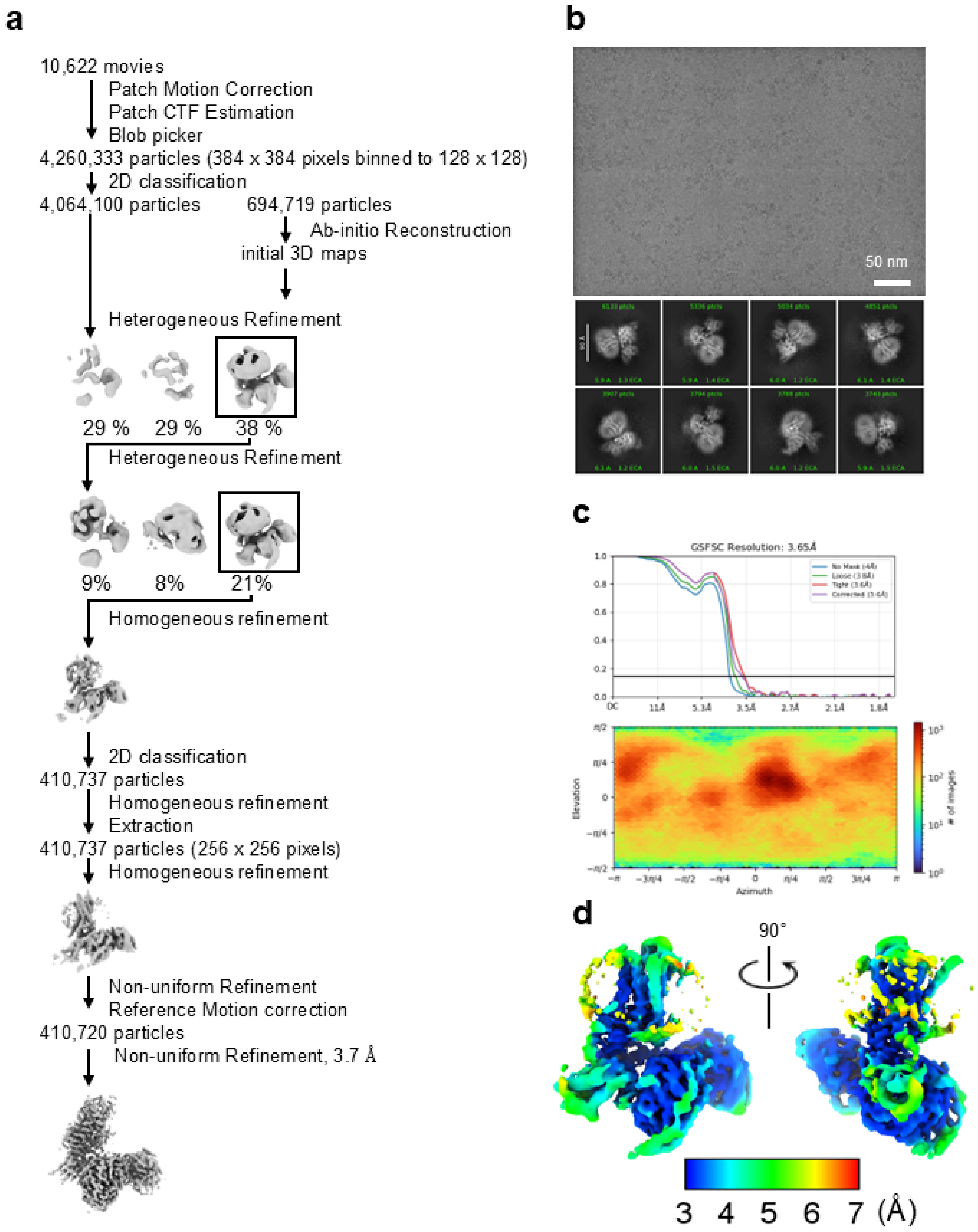

Fig. S13. Cryo-EM data processing of is-octβ₂R/oct. (a) Flowchart of data processing. (b) Representative micrograph and two-dimensional (2D) class averages. (c) Fourier shell correlation (FSC) curve and Euler angle distribution plot for the final refinement. (d) Final 3D density map colored by local resolution.

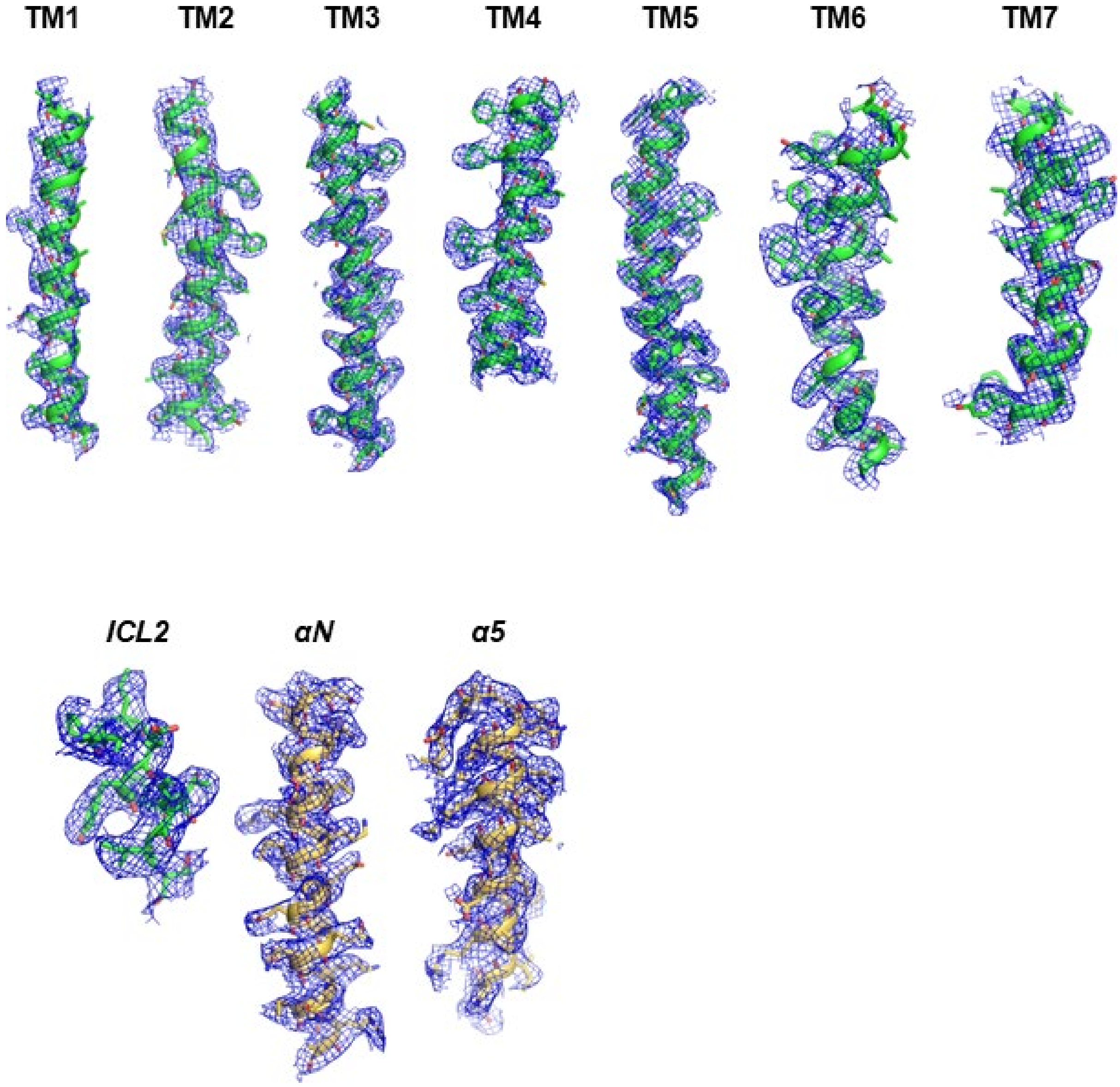

Fig. S14. Electron microscopy map about transmembrane regions of *I. scapularis* octβ_2R (is-octβ_2R)/2,4-dimethylphenylformamide (DPMF) and is-octβ_2R and Gs binding-related region.

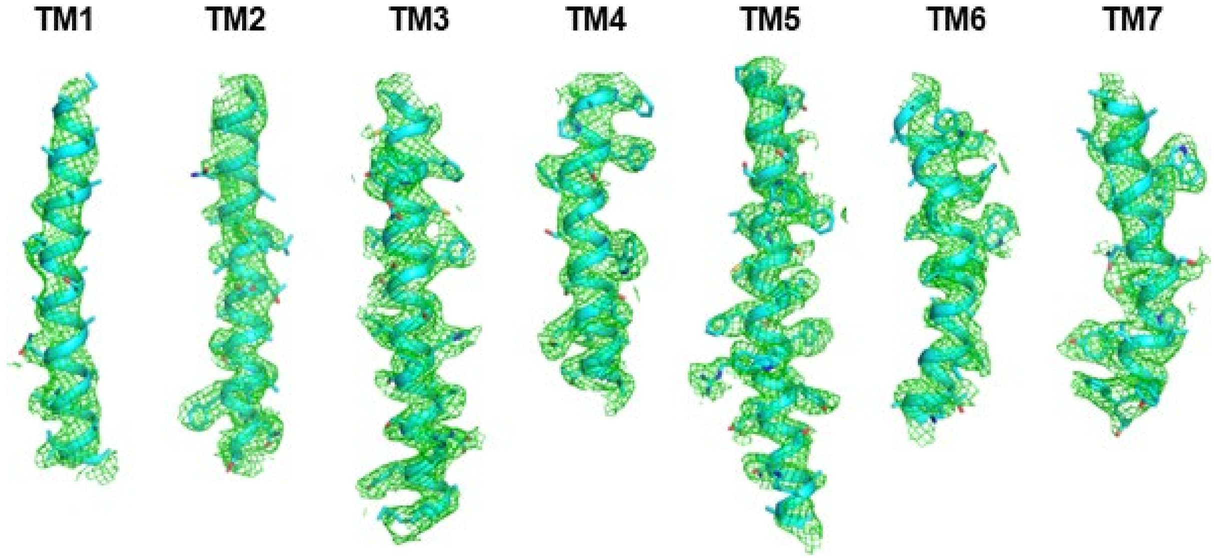

Fig. S15. Electron microscopy map about transmembrane regions of *I. scapularis* octβ_2R/oct.

a

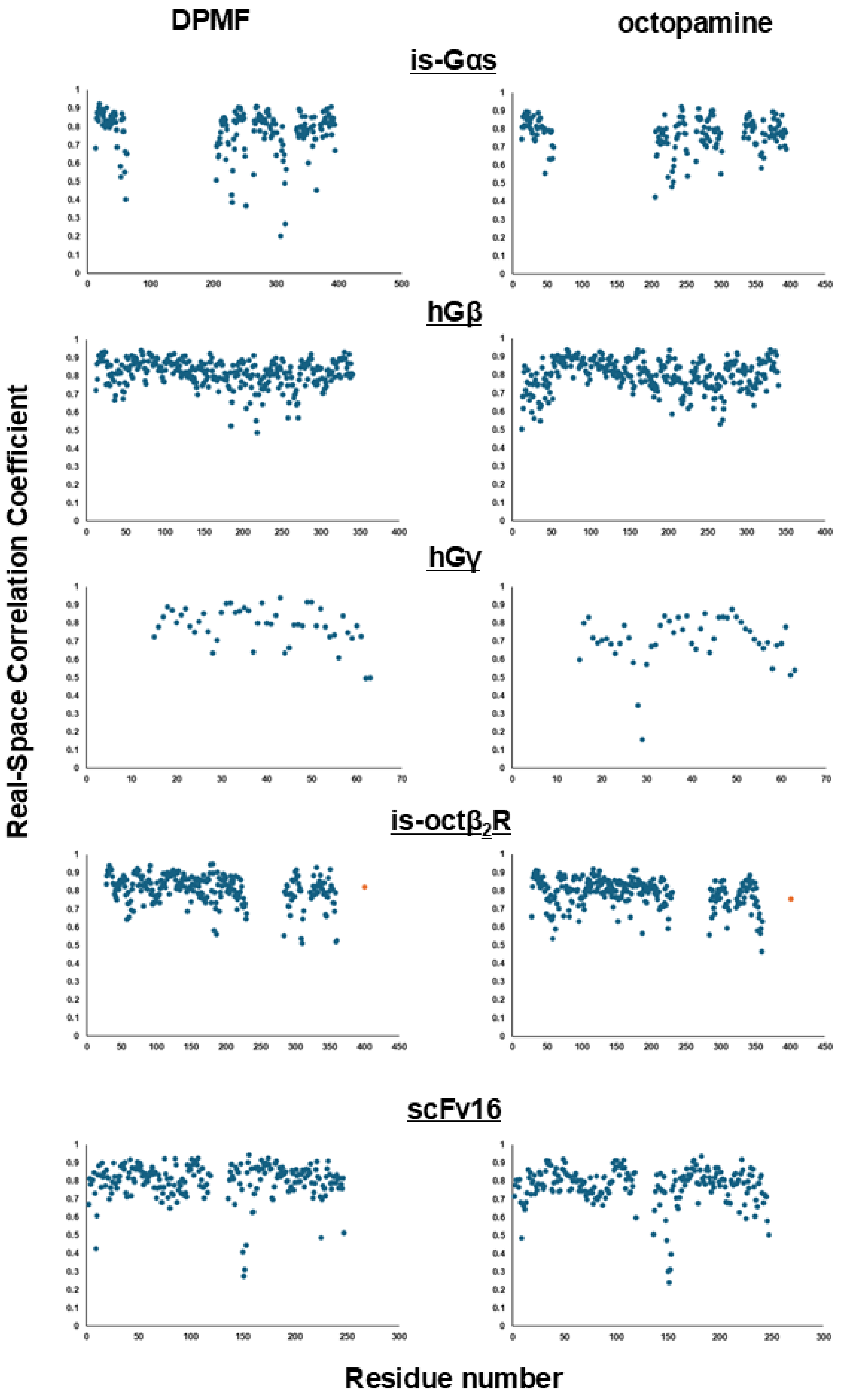

DPMF
octopamine
is-Gαs
hGβ
hGγ
is-octβ2R
scFv16
Real-Space Correlation Coefficient
Residue number

b

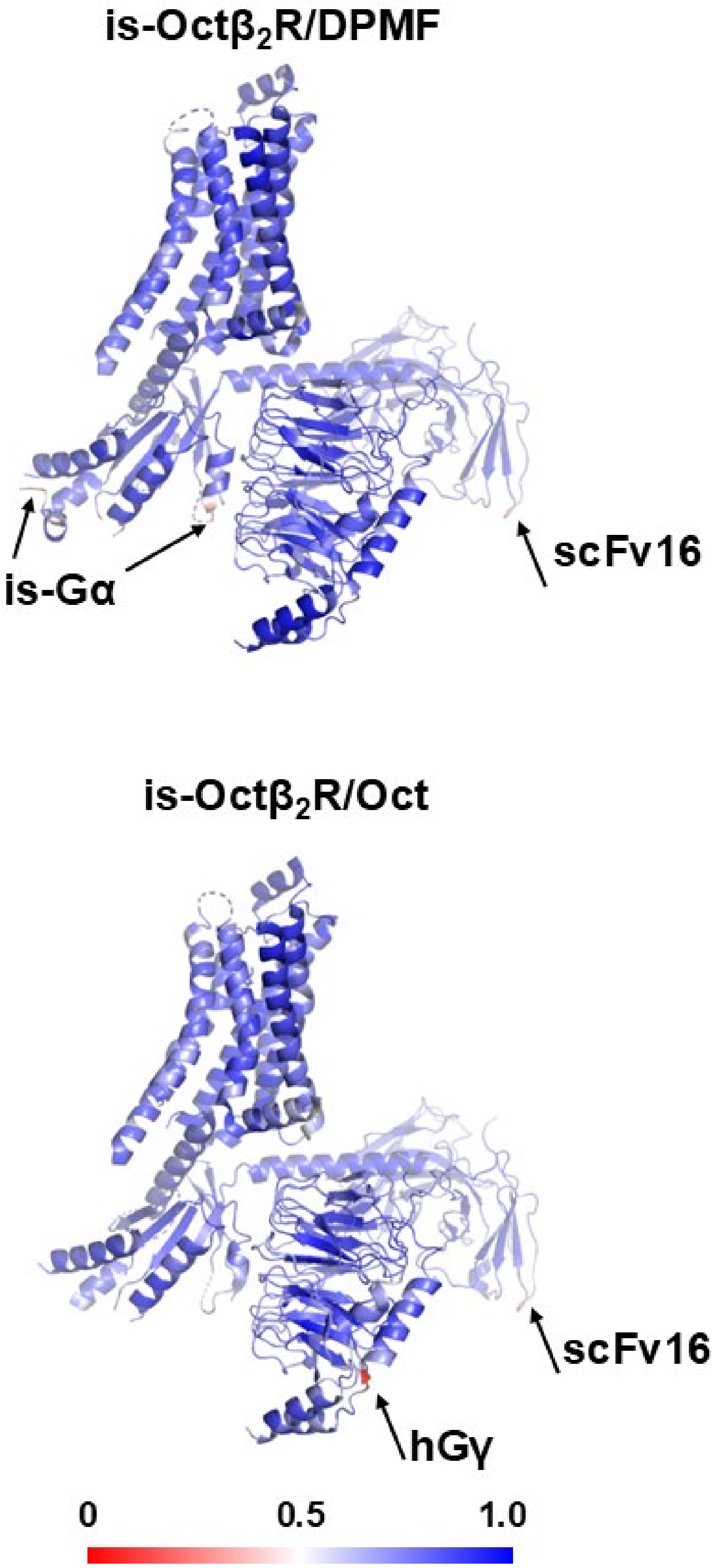

Fig. S16. Real-space correlation coefficient for is-Oct/agonist/G/scFv16 complexes. Real-space correlation coefficients (RSCCs) were calculated using Phenix. (a) Plot of RSCC per protein residue. Values for bound agonists are shown in orange. (b) Structures color-coded by RSCC. Regions with RSCC below 0.5, indicated by arrows, are not relevant to agonist and protein bindings.

Table S1. Amino acid conservation of residues around the orthosteric binding site among aminergic G-protein-coupled receptors

Residue	3.32	3.33	3.36	3.37	3.4	**34.52**	5.38	5.42	5.46	6.48	6.51	6.52	**6.55**	**7.39**	7.43
Conservation[*1]	D	V/I	C/V/S	T/S	I/S	**NC**	Y/F	S	S	W	F	F	**NC**	**NC**	Y/W
inv-octβ2R[*2] 503[*3]	D	V	S 464[*4]<br>T39	T 43<br>S 460	I	**F 482**<br>**W 21**	Y 498<br>F 5	S	S	W	F	F	**Y**	**F**	Y
inv-DR 699	D	I 458<br>V 211	S 656<br>C 12<br>A 1	T	I	**L 456**<br>**S 125**	Y	S	S	W	F	F	**N**	**T 385**<br>**V 176**<br>**S 102**[*5]	Y 487<br>W 182
inv-α_2AR 778	D	V 774<br>I 4	C 740<br>S 38	T	I	**L 627**<br>**I 85**<br>**V 64**	Y	S	S	W	F	F	**Y**	**F**	Y
inv-OAMB 275	D	V	C 273<br>S 2	T	I	**L 270**<br>**I 5**	Y	S 266<br>A 5	S	W	F	F	**Y**	**F**	Y
inv-TyrR 300	D	V 260<br>I 40	C	T	I	**L 296**<br>**P 4**	Y 282<br>F 18	S	S	W 299<br>F 1	F	F 299<br>S 1	**Y**	**T 282**<br>**V 14**	Y
mam-α_{1A}AR[*6] 1474	D	V	C 1455<br>S 19	T 1461<br>S 13	I	**I 1397**<br>**V 77**	Y	S	S	W	F	F	**L 781**<br>**M 691**	**F 1473**<br>**A 1**	Y
mam-α_{1B}AR 177	D	V	C	T	I	**V 127**<br>**I 50**	Y	S	S	W	F	F	**L**	**F**	Y
mam-α_{1D}AR 589	D	V	C	T 155<br>S 134	I	**I 583**<br>**V 6**	Y	S	S	W	F	F	**L 586**<br>**F 3**	**F**	Y
mam-α_{2A}AR 222	D	V 221<br>M 1	C	T	I	**I**	Y	S	S	W	F	F	**Y**	**F**	Y
mam-α_{2B}AR 258	D	V	C	T	I	**L 254**<br>**I 4**	Y	S	S	W	F	F	**Y**	**F**	Y
mam-α_{2C}AR 177	D	V	C	T	I	**L**	Y	S	S	W	F	F	**Y**	**F**	Y
mam-β_1AR 208	D	V	V	T	I	**F**	Y	S	S	W	F	F	**N**	**N**	Y
mam-β_2AR 312	D	V	V	T	I	**F**	Y	S	S	W	F	F	**N**	**N**	Y
mam-β_3AR 277	D 273<br>G 4	V 276<br>M 1	V	T	I	**F**	Y	S	S	W	F	F	**N 275**<br>**D 2**	**N**	Y 275<br>F 2
Residue	3.32	3.33	3.36	3.37	3.4	**34.52**	5.38	5.42	5.46	6.48	6.51	6.52	**6.55**	**7.39**	7.43
Conservation[*1]	D	V/I	C/V/S	T/S	I/S	**NC**	Y/F	S	S	W	F	F	**NC**	**NC**	Y/W
mam-DR1 399	D	I	C	S 397<br>C 2	S	**S 344**<br>**T 47**	Y 398<br>F 1	S	S	W	F	F	**N**	**V 382**<br>**I 11**	W

mam-DR2<br>949	D	V	C	T	I	**I<br>870<br>L<br>27<br>V<br>27**	F	S	S	W	F	F	**H**	**M<br>6<br>F<br>938<br>V<br>11**	G
mam-DR3<br>952	D	V	C	T	I	**S<br>947<br>A<br>5**	F	S	S	W	F	F	**H**	**T**	Y
mam-DR4<br>968	D	V	C	T	I	**K<br>444<br>Q<br>348<br>R<br>158**	Y<br>F	S	S	W	F	F	**H**	**T**	Y
mam-DR5<br>211	D	I	C	S<br>209<br>C<br>2	S	**S<br>210<br>A<br>1**	Y<br>210<br>F<br>1	S	S	W	F	F	**N**	**V**	W

*1: NC: not conserved.
*2: inv-: invertebrate.
*3: total number of receptors.
*4: number of receptors.
*5: Only the top three residues are shown.
*6: mam-: mammalian.

Table S2. Summary of EC_{50} and E_{max} values to I. scapularis-octβ_2R

	$logEC_{50}$	E_{max}
DPMF	-8.4 ± 0.1	24 ± 0.6
Octopamine	-7.7 ± 0.1	27 ± 0.7
Amitraz	-6.9 ± 0.1	26 ± 0.9
Tyramine	-5.8 ± 0.1	25 ± 1.7
Adrenaline	-5.5 ± 0.1	20 ± 2.1
Noradrenaline	-5.0 ± 0.3	32 ± 13.1
Dopamine	signal observed only at 10^{-5} M	
DMF	no activity	

Table S3 Amino acid conservation of octβ_2R from various species at mutation sites observed in amitraz-resistant strains

Residue	1.41	1.42	1.43	2.41	2.61	5.58
mutation	T60P	I61F	I62T	Y67S	N87S	Y215H
A	0	0	0	0	0	0
C	6	0	0	2	0	0
D	0	0	0	0	0	0
E	0	0	0	0	0	0
F	103	0	0	2	0	0
G	0	0	0	0	0	0
H	0	0	0	0	0	0
I	22	503	442	0	0	0
K	0	0	0	0	0	0
L	81	0	0	0	0	0
M	6	0	20	0	0	0
N	0	0	0	0	503	0
P	0	0	0	0	0	0
Q	0	0	0	0	0	0
R	0	0	0	1	0	0
S	185	0	0	2	0	0
T	51	0	0	0	0	0
V	11	0	41	0	0	0
W	38	0	0	0	0	0
Y	0	0	0	496	0	503
total	503	503	503	503	503	503

Amino acid sequences are obtained from a BLAST search using the non-redundant protein sequences database.

Table S4 Summary of assay results of homologous amitraz-resistant mutants

		$LogEC_{50}$	E_{max}
WT	amitraz	-7.5 ±0.1	17 ± 1
	DPMF	-8.1 ± 0.1	21 ± 1
	octopamine	-8.0 ± 0.1	23 ± 0.8
$T60^{1.41}P$	amitraz	-7.5 ± 0.2	17 ± 1.1
	DPMF	-8.1 ± 0.2	19 ± 1.2
	octopamine	-8.0 ± 0.1	19 ± 0.9
$I61^{1.42}F$	amitraz	-7.2 ± 0.2	14 ± 1
	DPMF	-8.1 ± 0.2	15 ± 1
	octopamine	-8.1 ± 0.1	19 ± 1.1
$I62^{1.43}T$	amitraz	-7.8 ± 0.1	18 ± 0.8
	DPMF	-8.7 ± 0.1	19 ± 0.5
	octopamine	-8.3 ± 0.1	21 ± 0.5
$Y67^{2.41}S$	amitraz	-7.2 ± 0.1	17 ± 0.7
	DPMF	-8.2 ± 0.1	19 ± 0.7
	octopamine	-7.9 ± 0.1	20 ± 0.7
$N87^{2.61}S$	amitraz	-7.3 ± 0.1	21 ± 0.8
	DPMF	-8.1 ± 0.1	23 ± 0.8
	octopamine	-8.0 ± 0.1	23 ± 0.7
$Y215^{5.58}H$	amitraz	-7.2 ± 0.2	8 ± 0.6
	DPMF	-8.2 ± 0.2	10 ± 0.7
	octopamine	-7.8 ± 0.2	12 ± 0.7

Table S5. Amino acid conservation of Y$^{5.58}$ of *I. scapularis* and human GPCRs

GPCR from *Ixodes scapularis*[*1]			5.50								5.58			
is-octβ2R	W	I	P	C	C	I	M	L	F	T	Y	W	R	I
dopamine_D2-like_recepto	Y	I	P	C	I	V	M	V	F	L	Y	Y	K	I
dopamine_receptor_2	Y	A	P	L	M	V	M	V	F	T	Y	Y	R	I
dopamine_receptor_1	Y	M	P	C	V	V	M	V	A	L	Y	T	R	L
tyramine_receptor_1	F	A	P	M	F	I	M	T	I	V	Y	F	K	I
alpha-2_adrenergic_receptor	Y	V	P	A	V	V	M	V	F	V	Y	I	R	I
serotonin_receptor (5-HT2B)	Y	V	P	L	I	M	I	L	L	L	Y	W	R	I
serotonin_receptor (5-HT2A)	Y	V	P	L	I	L	I	L	L	L	Y	W	R	I
probable_G-protein_coupled_receptor_No9	Y	I	P	M	L	F	M	L	F	F	N	Y	R	I
trissin receptor	F	I	P	L	A	I	M	A	V	M	Y	S	I	I
trissin receptor	L	V	P	L	I	I	I	T	V	L	Y	T	I	I
allatostatin receptor	V	V	P	L	A	L	V	F	I	L	Y	V	L	M
tachykinin-like_peptides_receptor	F	L	P	L	C	V	I	C	Y	T	Y	G	R	I
neuropeptide_FF_receptor	L	L	P	L	T	I	V	C	Y	T	Y	G	R	I
Ryamide receptor	F	F	P	L	L	A	L	I	F	T	Y	T	R	I
neuropeptide_SIFamide_recepto	L	L	P	L	C	V	I	T	L	C	Y	I	F	I
SIFamide_recepto	L	F	P	L	L	L	I	I	V	C	Y	S	C	I
gonadotropin-releasing_hormone_II	G	V	P	L	S	A	I	L	V	C	Y	S	R	I
ACP-R5	G	L	P	L	A	A	I	V	I	C	Y	S	R	I
cardioacceleratory_peptide_receptor	V	V	P	G	L	L	I	S	A	F	Y	L	S	L
oxytocin receptor-like	V	V	P	G	L	L	I	S	A	F	Y	L	S	L
G-protein_coupled_receptor_moody	F	L	P	T	V	F	F	V	V	C	Y	S	R	I
neuropeptide_Y_receptor_type_2	V	V	P	F	G	I	I	S	F	C	Y	M	R	V
gonadotropin-releasing_hormone_receptor	L	L	P	L	V	T	L	I	T	T	Y	I	C	T
muscarinic_acetylcholine_receptor_DM1	Y	V	P	V	T	V	M	C	I	L	Y	W	R	I
orexin_receptor_type_2	V	A	P	F	A	L	M	S	A	V	Y	Y	Q	M
QRFP-like_peptide_receptor	C	I	P	T	I	V	M	G	Y	A	Y	T	R	I
muscarinic_acetylcholine_receptor_M5	Y	A	T	L	V	V	L	F	I	L	Y	G	G	I
cardioacceleratory_peptide_receptor	F	V	P	A	L	V	I	T	A	C	Y	S	V	I
adenosine_receptor_A2a	W	S	C	T	L	V	T	L	S	L	Y	F	F	V
dopamine_D2-like_receptor	F	V	P	L	I	V	M	G	F	C	Y	F	N	I
tachykinin-like_peptides_receptor_86C	I	F	P	V	V	T	M	A	A	T	Y	A	R	M
oxytocin_receptor	F	V	P	L	M	V	L	T	F	T	Y	V	C	I
QRFP-like_peptide_receptor	F	V	P	V	F	V	M	S	S	A	Y	A	L	I
pyrokinin-1_receptor	L	L	P	M	S	V	I	L	V	L	Y	V	C	I
adenosine_A3_receptor	V	P	S	V	L	T	V	I	Y	T	Y	F	F	I
adenosine_receptor_A1	F	L	P	L	V	L	M	C	A	I	Y	A	H	I
Human GPCRs			5.50								5.58			
negatively charged	0	0	0	0	0	0	0	0	0	0	2	1	6	1
positively charged	0	0	0	1	0	0	0	0	2	0	0	2	96	3
hydrophilic	50	11	19	34	47	29	40	71	54	147	236	96	49	27
hydrophobic	224	263	255	239	227	245	234	203	218	127	36	175	123	243

*1 Protein ID, is-octβ2R: XP_029841166.1, dopamine_D2-like_receptor: XP_042145760.1, dopamine_receptor_2: XP_002399655.1, dopamine_receptor_1: XP_042142135.1, tyramine_receptor_1: XP_002415939.3, alpha-2_adrenergic_receptor: XP_029831528.1, serotonin_receptor (5-HT2B): EEC15134.1, serotonin_receptor (5-HT2A): EEC03313.1, probable_G-protein_coupled_receptor_No9: XP_042143173.1, trissin receptor: XP_029847302.2, trissin receptor: XP_040062527.2, allatostatin receptor: EEC00437.1, tachykinin-like_peptides_receptor: XP_029838406.1, neuropeptide_FF_receptor: EEC16965.1, Ryamide receptor: XP_040075532.1, neuropeptide_SIFamide_receptor: XP_029828900.2, SIFamide_receptor: AGE11606.1, gonadotropin-releasing_hormone_II: XP_029845158.4, ACP-R5: AHE76188.1, cardioacceleratory_peptide_receptor: XP_040069955.1, oxytocin receptor-like: XP_040069958.2, G-protein_coupled_receptor_moody: XP_042144550.1, neuropeptide_Y_receptor_type_2: XP_042143278.1, gonadotropin-releasing_hormone_receptor: XP_040079665.2, muscarinic_acetylcholine_receptor_DM1: XP_002403135.1, orexin_receptor_type_2: XP_002416435.4, QRFP-like_peptide_receptor: XP_029837242.1, muscarinic_acetylcholine_receptor_M5: XP_002416160.3, cardioacceleratory_peptide_receptor: XP_029849542.1, adenosine_receptor_A2a: XP_029836549.2, dopamine_D2-like_receptor: XP_029851696.2, tachykinin-like_peptides_receptor_86C: XP_040066851.1, oxytocin_receptor: XP_029841441.2, QRFP-like_peptide_receptor: XP_042148921.1, pyrokinin-1_receptor: XP_042145382.1, adenosine_A3_receptor: EEC03285.1, adenosine_receptor_A1: XP_002400568.1

Table S6. Summary of mutant assay results and interactions for residues associated with ligand binding

Mutant	Ligand	$logEC_{50}$	E_{max}	Interaction
D110$^{3.32}$A	Octopamine	no activity		
	DPMF	no activity		salt bridge
D110$^{3.32}$N	Octopamine	no activity		
	DPMF	no activity		
V111$^{3.33}$A	Octopamine	signal observed only at 10^{-5} M		CH-π
	DPMF	-6.6 ± 0.1	25 ± 1.1	
S114$^{3.36}$A	Octopamine	signal observed only at 10^{-5} M		hydrogen bond
	DPMF	-6.6 ± 0.1	23 ± 1.2	
T115$^{3.37}$A	Octopamine	signal observed only at 10^{-5} M		(S203)
	DPMF	-8.3 ± 0.1	23 ± 1.0	
I118$^{3.40}$A	Octopamine	no activity		
	DPMF	no activity		
F189$^{45.52}$A	Octopamine	no activity		
	DPMF	no activity		edge-to-edge
Y195$^{5.38}$A	Octopamine	no activity		
	DPMF	no activity		
S199$^{5.42}$A	Octopamine	signal observed only at 10^{-5} M		hydrogen bond
	DPMF	signal observed only at 10^{-5} M		van der Waals
S203$^{5.46}$A	Octopamine	signal observed only at 10^{-5} M		hydrogen bond
	DPMF	-8.9 ± 0.2	22 ± 1.5	
W300$^{6.48}$A	Octopamine	no activity		
	DPMF	no activity		
F303$^{6.51}$A	Octopamine	no activity		edge-to-π
	DPMF	no activity		van der Waals
F304$^{6.52}$A	Octopamine	-6.4 ± 0.2	21 ± 2	
	DPMF	-7.2 ± 0.1	16 ± 0.8	
Y307$^{6.55}$A	Octopamine	no activity		edge-to-edge
	DPMF	no activity		π-π
F328$^{7.39}$A	Octopamine	-6.0 ± 0.1	26 ± 1.7	π-hydrogen
	DPMF	no activity		CH-π
Y332$^{7.43}$A	Octopamine	signal observed only at 10^{-5} M		(D110)
	DPMF	signal observed only at 10^{-5} M		van der Waals
WT	Octopamine	-7.6 ± 0.1	27 ± 1.5	
DPMF	-8.1 ± 0.1	25 ± 0.7		

Table S7. Distance of transmembrane (Å)

	octβ_2R/DPMF	octβ_2R/oct	β_2AR(ina)
TM3–TM5	13.7	13.4	13.6
TM3–TM6	15.8	15.2	15.4
TM3–TM7	13.8	13.0	13.6
TM5–TM6	12.2	12.4	12.5
TM5–TM7	18.2	17.6	17.6
TM6–TM7	9.1	8.6	8.0

Distances are measured among the centers of gravity of the Cα atoms of residues 3.31–3.33, 5.41–5.43, 6.50–6.52, and 7.38–7.40 in transmembrane (TM) 3, 5, 6, and 7, respectively.

Table S8. Amino acid conservation of residues 6.55 and 7.39

Residue	6.55	7.39
(1) invertebrate α_2AR, OAMB, and octβ_2R / mammalian α_2ARs		
	Y	F
inv-α_2AR	Y (778)	F (778)
inv-OAMB	Y (275)	F (275)
inv-octβ_2R	Y (503)	F (503)
mam-α_{2A}AR	Y (222)	F (222)
mam-α_{2B}AR	Y (258)	F (258)
mam-α_{2C}AR	Y (177)	F (177)
(2) invertebrate inv- α_1AR / mammalian α_1AR		
	L/M	F
inv- α1AR	L (156)	F (130)
	Y (12)	W (42)
mam-α_{1A}AR	L (781)	F (1473)
	M (691)	A (1)
mam-α_{1B}AR	L (177)	F (177)
mam-α_{1D}AR	L (586)	F (589)
	F (3)	
(3) mammalian βARs		
	N	N
mam-β_1AR	N (208)	N (208)
mam-β_2AR	N (312)	N (312)
mam-β_3AR	N (275)	N (277)
	D (2)	
(4) invertebrate DRs / mammalian TAAR1, DR1, and DR4		
	Hydrophilic	Not conserved
inv-DR	N (669)	T (385)
		V (176)
		S (176)
mam-DR1	N (399)	V (382)
		I (11)
		M (6)
mam-DR4	H (968)	T (968)
(5) invertebrate TyrR, DRs / mammalian TAAR1, DR1, and DR4		
	Y	Not conserved
inv-TyrR	Y (300)	T (282)
		V (14)
		I (4)
(6) mammalian TAAR1		
	Not conserved	Not conserved
mam-TAAR1	T (688)	V (607)
	N (119)	I (189)
	M (97)	L (73)
	L (29)	F (37)
	I (16)	
